# Simultaneous determination of 114 pesticides in complex Chinese herbal medicine *Fritillaria* using ordered mesoporous carbon CMK-3 as a reversed-dispersive solid phase extraction sorbent†

**DOI:** 10.1039/d0ra07229j

**Published:** 2021-01-20

**Authors:** Tong Wu, Peipei Qi, Jiao Wang, Zhiwei Wang, Shanshan Di, Hao Xu, Huiyu Zhao, Changshan Zhao, Xinquan Wang

**Affiliations:** College of Agriculture, Northeast Agricultural University No. 600 Changjiang Road Harbin 150030 P. R. China csz-hlj@sohu.com +86 451 55191775; State Key Laboratory for Managing Biotic and Chemical Threats to the Quality and Safety of Agro-products, Institute of Quality and Standard of Agro-products, Zhejiang Academy of Agricultural Sciences Hangzhou 310021 P. R. China wangxinquan212@163.com +86 571 86419051; Agricultural Ministry Key Laboratory for Pesticide Residue Detection Hangzhou 310021 P. R. China; Key Laboratory of Detection for Pesticide Residue and Control of Zhejiang Hangzhou 310021 P. R. China

## Abstract

*Fritillaria*, a traditional Chinese herbal medicine, is classified into many medicinal species and contains numerous complex components. It is thus difficult to simultaneously detect multiple pesticide residues in different *Fritillaria* species. An easy, reliable, and widely applicable analytical method based on a modified Quick, Easy, Cheap, Effective, Rugged, and Safe (QuEChERS) method coupled with LC-MS/MS was developed to solve these problems encountered during pesticide residue analysis in complex *Fritillaria* matrices. Ordered mesoporous carbon CMK-3 and a primary secondary amine (PSA) were used as efficient purification sorbents by optimization of the QuEChERS process. Systematic method validation was performed for four species of *Fritillaria*. The matrix effect of pesticides varied among different *Fritillaria* species, and matrix-matched standard solutions were thus employed for quantitative analysis. The mean recoveries of all pesticides ranged from 88.6% to 95.5%, with mean relative standard deviations (RSD) lower than 6% at spiked concentrations of 30, 120, and 240 μg kg^−1^. The limits of quantification (LOQ) for the developed method were in the range of 30–120 μg kg^−1^. This method was further used to analyze 47 *Fritillaria* samples from Zhejiang province, China, and seven pesticides were detected in 22 *Fritillaria* samples. These results demonstrate that the developed method is suitable for an accurate analysis of multiple pesticide residues in various *Fritillaria*.

## Abstract 2

*Fritillaria*, a traditional Chinese herbal medicine, is classified into many medicinal species and contains numerous complex components.

## Graphical abstract

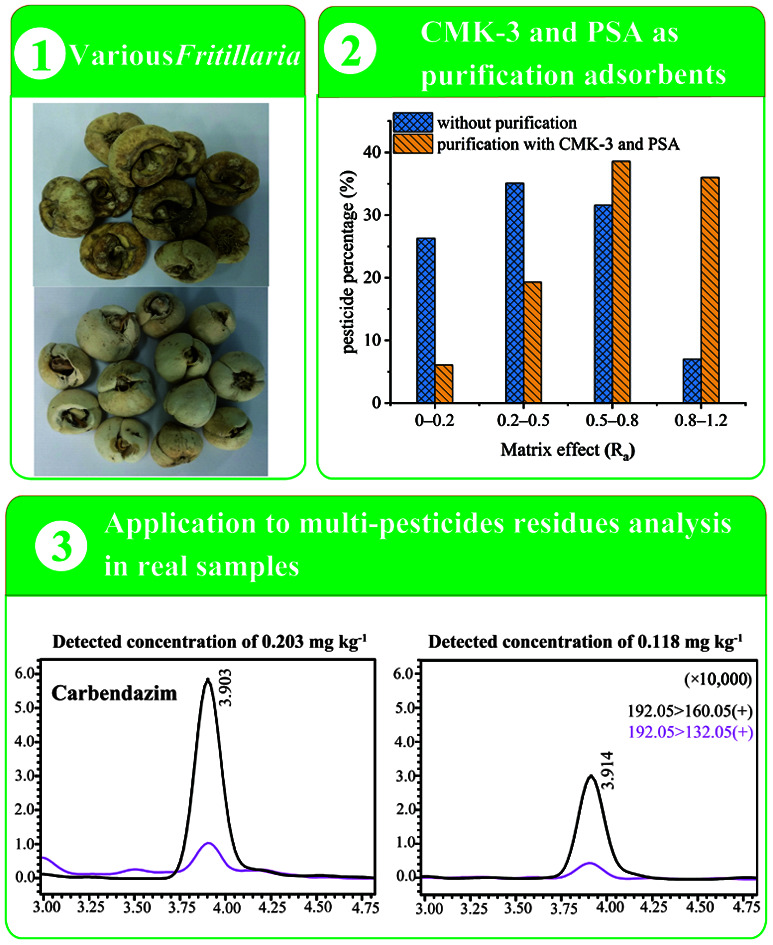

## 1. Introduction

*Fritillaria*, an important traditional Chinese herbal medicine with antitussive and expectorant properties, is widely used as an ingredient in patented medicines (herbal products) and health foods. Currently, there are more than 200 different Chinese patented medicines containing *Fritillaria* for the treatment of cough and phlegm.^1^ The expanding demand for herbal products has led to an increase in the cultivation area and export volume of *Fritillaria*.^2^ To improve the yield and quality of *Fritillaria*, pesticides are inevitably used to prevent pests (*e.g.*, grub, cutworm), diseases (*e.g.*, gray mold, black spot), and weeds during cultivation. However, only eight pesticides are currently registered for use on *Fritillaria* in China. These eight pesticides are not enough to prevent all of the diseases and pests on *Fritillaria*. Therefore, some pesticides that are not registered for *Fritillaria* cultivation are used,^3^ the scientific dosage standards of which are not set for use on *Fritillaria*. The abuse of pesticides often occurs when unregistered pesticides are used by growers, and the abuse of these pesticides in China has led to large amounts of multiple pesticide residues in *Fritillaria*, which have been confirmed by previous reports.^3,4^

Considering that pesticide residues in herbal medicines may pose potential health risks to consumers, some countries and organizations have established maximum residue limit (MRL) regulations for these medicinal plants. While China has issued MRLs of 21 pesticides in several herbs such as ginseng,^5^ the European Union (EU) has set more than 400 MRLs for herbal infusions.^6^ However, there are no detailed regulations regarding the MRLs for *Fritillaria*, and the risk of pesticide residues in *Fritillaria* can only be assessed on the basis of existing MRLs for similar herbs.^7^ There are differences in the MRL standards established by different countries or organizations, which poses an obstacle to the assessment of pesticide residue risk in *Fritillaria* and the export of its products. Therefore, it is necessary to develop an efficient and reliable method for the simultaneous detection of multiple pesticide residues in *Fritillaria*, and monitor and assess the associated risks.

*Fritillaria* contains various active components, such as steroidal alkaloids, fatty acids, terpenoids, saponins, amino acids, polysaccharides,^8,9^ which can interfere with the quantification of target analytes. According to the records of the Chinese Pharmacopeia, there are many *Fritillaria* species with pharmacological effects, and the types and contents of the active components vary with the species. For example, *Fritillaria cirrhosa* D.Don (FC) and *F. ussuriensis* Maxim. (FU) contain imperialine (an isosteroidal alkaloids), but *F. thunbergii* Miq. (FT) does not contain imperialine. Meanwhile, FC contains more imperialine than FU.^10^ Thus, it is a great challenge to determine the residue levels of multiple pesticides simultaneously in the different *Fritillaria* species. Previous studies have focused only on several kinds of pesticides and the individual specie of *Fritillaria*,^11,12^ as such, there is no comprehensive method to determine multiple pesticides in different species of *Fritillaria*. The development of a high-throughput detection method for various *Fritillaria* is essential for monitoring the quality of these *Fritillaria* and protecting consumer safety.

Sample preparation is essential to ensure accurate quantification of target analytes in pesticide residue analysis. In previous reports, accelerated solvent extraction,^13^ solid phase extraction,^14,15^ and Quick, Easy, Cheap, Effective, Rugged, and Safe (QuEChERS) method^4,16,17^ have been used for the preparation of *Fritillaria* samples. Compared to the other methods, the QuEChERS method has advantages such as simplified operation steps and reduced solvent consumption. Moreover, the QuEChERS method has demonstrated wide application during multiple pesticide residues analysis in complex matrices.^18,19^ Therefore, the aim of the present study is to establish a rapid, widely applicable detection method based on QuEChERS for multiple pesticides, and solve the practical problem of pesticide residue determination in different *Fritillaria* species.

The typical QuEChERS method includes acetonitrile extraction and purification by dispersive solid phase extraction (dSPE).^20^ The dSPE purification procedure removes interferences from the matrix by cleanup sorbents, and the retained target analytes can be accurately quantified.^21,22^ Thus, the application of a suitable sorbent plays a key role in effectively removing the matrix interferences during dSPE. Ordered mesoporous carbon is a new type of carbonaceous material with a high surface area, large pore volume, and ordered structure. Ordered mesoporous carbon and its functionalized materials have been successfully used as sorbents for the removal of phenol,^23^ antibiotics,^24^ proteins,^25^ dyes^26^ and heavy metals,^27^ and for the adsorption and enrichment of some pesticides from water samples.^28,29^ Moreover, the excellent adsorption capacity of ordered mesoporous carbons for alkaloids has also been reported.^30^ Based on these studies, we were inspired to consider that ordered mesoporous carbon could be used as a sorbent to remove interferences, such as steroidal alkaloids, from the *Fritillaria* matrix. Hence, ordered mesoporous carbon CMK-3 was selected as the purification sorbent, because it is commercially available and has a high adsorption rate and adsorption capacity.^23,31^

The overall aim of this study was to develop an easy, rapid, and reliable QuEChERS method using CMK-3 as a sorbent for simultaneous determination of multiple pesticide residues in different *Fritillaria* species by LC-MS/MS. A total of 114 common pesticides that may be used in *Fritillaria* cultivation were selected as target analytes, and the recovery and matrix effect of all pesticides were used as a guide to select the optimal extraction and purification conditions. The optimized method was used to detect pesticide residue levels in 47 *Fritillaria* samples from Zhejiang province, China.

## 2. Experimental

### 2.1. Materials and chemicals

The four medicinal species of *Fritillaria* from various areas of China were used in the experiment, including FT from Zhejiang province, FU from Liaoning province, FC from Sichuan province, *Fritillaria delavayi* Franch. (FD) from Tibet autonomous region. These samples were analyzed and proved to be uncontaminated. The FT samples were used during the method optimization. CMK-3 was obtained from Nanjing XFNANO Materials Tech Co. Ltd. (Nanjing, China), and the primary secondary amine (PSA) was obtained from Agilent (DE, USA). Anhydrous magnesium sulfate (MgSO_4_) and sodium chloride (NaCl) were obtained from Bonna-Agela Technologies Co. Ltd. (Tianjin, China).

A total of 114 pesticide standards were purchased from the Ministry of Agriculture (Tianjin, China), Agro-Environmental Protection Institute, and Shanghai Pesticide Research Institute (Shanghai, China). A mixed standard solution of 114 pesticides at a concentration of 5.0 mg L^−1^ was prepared in methanol and stored at 4 °C. HPLC-grade acetonitrile (ACN) and methanol were supplied by Merck (New Jersey, USA). HPLC-grade ammonium formate was provided by Tedia (Fairfield, USA). Purified water was prepared by a Millipore Milli-Q system. Acetic acid (HAC) was an analytical grade reagent.

### 2.2. Sample preparation

The *Fritillaria* samples were ground and stored at room temperature. *Fritillaria* powder (2 g) was weighed into a 50 mL centrifuge tube, and 5 mL water was added. After mixing for 1 min, 6 mL acetonitrile was added and the mixture was vortexed for 1 min. Next, NaCl (1.5 g) and anhydrous MgSO_4_ (6 g) were added and after vortexing for 1 min, the mixtures were centrifuged at 7000 rpm for 5 min.

After centrifugation, 1 mL of the supernatant acetonitrile phase were collected and transferred into a 2 mL centrifuge tube containing 5 mg of CMK-3, 30 mg of PSA and 150 mg of anhydrous MgSO_4_. The mixtures were vortexed for 1 min and then centrifuged at 7000 rpm for 5 min. Next, 0.5 mL of the supernatants were mixed with 0.5 mL of water, and filtered through 0.22 μm filters for analysis by LC-MS/MS.

In addition, an appropriate volume of the mixed standard solution was evenly dropped into *Fritillaria* powder (2 g) for method optimization and recovery experiments. After standing for 1 h, the spiked samples were extracted and purified according to the above procedures.

### 2.3. LC-MS/MS analysis

The LC-MS/MS system consisted of Nexera X2 LC-30AD (Shimadzu, Kyoto, Japan) and a Shimadzu 8050 triple-quadrupole mass spectrometer (Shimadzu, Kyoto, Japan). A chromatographic column ACE Excel 2 C18 (100 mm × 2.1 mm, 2 μm) was used for the separation of the target pesticides and maintained at 35 °C. The mobile phases were 5 mmol L^−1^ ammonium formate aqueous solution (A) and pure methanol (B) at a flow rate of 0.3 mL min^−1^. The gradient elution program was presented as follows: 0–1.00 min, 40% B; 1.00–3.00 min, 40–80% B; 3.00–5.00 min, 80–95% B; 5.00–8.00 min, 95% B; 8.00–8.01 min, 95–5% B; 8.01–10.00 min, 5% B. The total run time was 10 min and the injection volume was 2 μL.

The MS/MS detection was performed using electrospray ionization and multiple reaction monitoring mode. The operation parameters were as follows: capillary voltage, 4000 V and −3000 V for the positive and negative ion modes, respectively; capillary temperature, 300 °C; flow rate of the nebulizing gas (nitrogen, 99.9% purity), 3 L min^−1^; flow rate of the drying gas (nitrogen, 99.9% purity), 10 L min^−1^; collision gas, argon. Table S1† lists the precursor ions, product ions, collision energies, and deviations for all the target pesticides.

### 2.4. Validation study

The developed method was validated for four *Fritillaria* species, namely, FT, FU, FC, FD. The linearity, matrix effect, recovery, precision, limit of detection (LOD) and limit of quantification (LOQ) of the developed method were evaluated.

#### 2.4.1. Linearity validation

The calibration curves for all of the pesticides were built by matrix-matched standard solutions and solvent standard solutions at concentrations of 1, 2, 5, 10, 20, 50, 100, and 250 μg L^−1^. Matrix-matched standard solutions of eight concentration levels were prepared in blank matrix extraction solutions/water (1 : 1, v/v), and solvent standard solutions were prepared in ACN/water (1 : 1, v/v). The linear regression equations and correlation coefficients (*R*) of the calibration curves were calculated by plotting the pesticide peak area *versus* concentration. The *R* was higher than 0.99, indicating good linearity of pesticides.

#### 2.4.2. Matrix effect validation

The matrix effect represented the interference of the co-extracted compounds from the sample on the detection result of the target analytes, which is usually shown to enhance or inhibit the response of the target analytes. The slope ratio of the matrix-matched calibration curve to solvent calibration curve was calculated to evaluate the matrix effect (ME_s_)^32^ of four *Fritillaria* samples, and the calculation equation is as follows:1
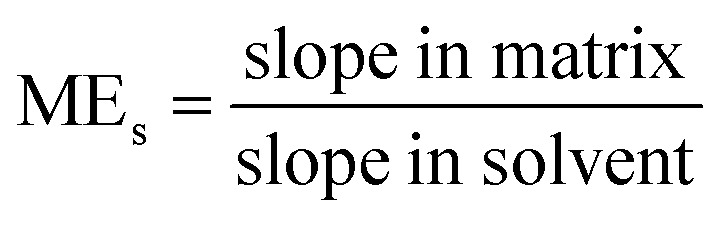


For the investigation of the adsorbent purification effect in Section 3.2, the peak area ratio of pesticides in matrix-matched standard solution *versus* those in solvent standard solution at the same concentration was used to evaluate the matrix effect (ME_p_),^33^ because it was conducive to more visually display the improvement degree of the matrix effect after using the purification adsorbents, and the calculation equation is as follows:2
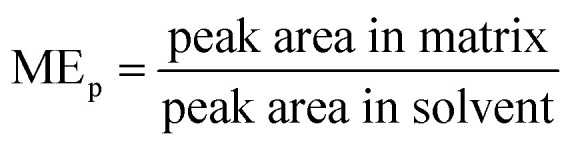


The values of ME_s_ and ME_p_ closer to 1 represent the weaker matrix effect. The values of ME_s_ and ME_p_ less than 1 or greater than 1 indicate matrix suppression or matrix enhancement, respectively.

#### 2.4.3. Recovery and precision validation

The recovery was calculated by the percentage of measured concentration from the spiked sample to the spiked concentration. For the recovery experiments, the four *Fritillaria* samples were spiked at concentrations of 30, 120, and 240 μg kg^−1^ with three replicates for each concentration. The precision reflected the consistency of analysis results of different batches under the same conditions, and was usually assessed using relative standard deviation (RSD) of the recovery for each pesticide. According to the European guidance document SANTE/12682/2019, the recoveries should be in the range of 70–120% associated with RSD below 20%, but broader recoveries in the range of 60–130% with good precision are considered acceptable for multi-residues analysis.^34^

#### 2.4.4. LOD and LOQ validation

The LOD was defined as the lowest concentration at which the target analytes can be identified by the instrument. In this work, the LOD of each target pesticide from four *Fritillaria* matrices was examined for the instrument. The instrument LOD was determined as the concentration providing the response of three times the signal-to-noise ratio,^35^ which was estimated by the instrument based on the response of matrix-matched standard solutions from four *Fritillaria* at 1 μg L^−1^. The LOQ was defined as the lowest concentration at which the target analytes can be accurately quantified. In this work, the method LOQ of each target pesticide in the four *Fritillaria* samples was investigated to ensure the accuracy and reliability of this method in practice. The method LOQ was determined as the lowest spiked concentration that met the recovery and precision criterion in the recovery experiment.

## 3. Results and discussion

### 3.1. Optimization of the extraction solvent

The selection of extraction solvent is the first step to be overcome in multi-residue analysis. ACN was widely used as the extraction solvent because of its satisfactory extraction effect for most pesticides.^36^ It has been reported that adding acid to the extraction solvent has a positive influence on the extraction efficiency and stability of base-sensitive pesticides in multi-residue analysis.^37,38^ Thus, the extraction effects of ACN, 0.5% HAC in ACN, and 1.0% HAC in ACN were evaluated.

**Fig. 1:**
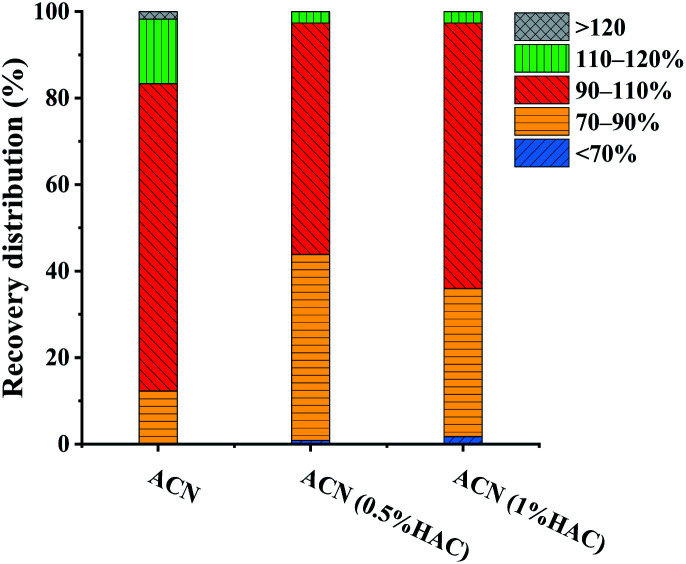
Influence of extraction solvent (ACN, ACN containing 0.5% HAC or ACN containing 1.0%) on the recovery distribution of the pesticides (extracted from the FT sample spiked at 120 μg kg^−1^).

All 114 pesticides were spiked to the FT samples at the concentration of 120 μg kg^−1^, and extracted with 6 mL of different extraction solutions (ACN, 0.5% HAC in ACN, or 1.0% HAC in ACN). The extracted supernatants were directly injected into LC-MS/MS for analysis without a purification step. The recoveries for each pesticide were calculated, and the overall recovery distributions of the pesticides were evaluated. As shown in Fig. 1, it was observed that there were no significant differences in the recoveries using different extraction solvents, with over 98.2% of the pesticide recoveries in the range of 70–120%. This indicated that all the extraction solvents achieved satisfactory extraction effects. However, further observation of the pesticide recovery distributions revealed some differences. When ACN was used as the extraction solvent, all pesticide recoveries were larger than 70%, and 71.1% of the pesticide recoveries were in the range of 90–110%. However, only 53.5% and 61.4% of the pesticide recoveries were between 90–110% using 0.5% HAC in ACN and 1.0% HAC in ACN, respectively. Taking both convenient extraction process and extraction efficiency into consideration, ACN was chosen as the optimum extraction solvent for further experiments.

### 3.2. Optimization of the sorbent amount

*Fritillaria* mainly contains steroidal alkaloids, fatty acids, and other active components, yet these active components are the main interference factors in multi-pesticide residue analysis, which makes the sample matrix complicated. Therefore, the focus of this experiment was to select efficient purification sorbents and remove the interferences in the sample matrix based on the QuEChERS method. CMK-3 was selected as a sorbent to remove alkaloids, and PSA was selected to remove polar substances such as fatty acids in the sample matrix.^39,40^ The amounts of CMK-3 and PSA were optimized to obtain the most efficient purification effect and pesticide recoveries. In the following optimization experiments, 6 mL of ACN was used to extract the analytes in the spiked FT samples at a concentration of 120 μg kg^−1^, and 1 mL of the obtained extraction solutions was drawn for purification analysis.

#### 3.2.1. Dosage of CMK-3

**Fig. 2:**
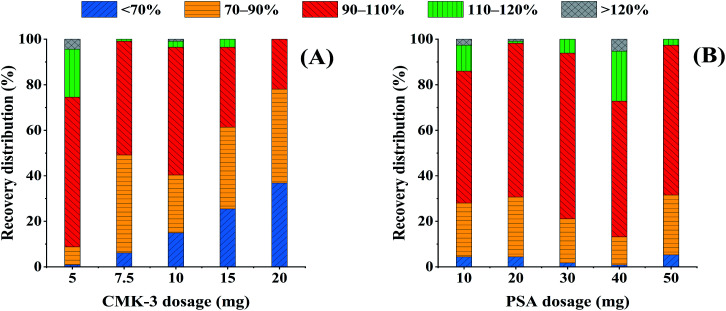
Effect of two adsorbent dosages on the recovery distribution of the pesticides (extracted from the FT sample spiked at 120 μg kg^−1^). (A) The amount of CMK-3 (5, 7.5, 10, 15, or 20 mg); (B) the amount of PSA (5 mg of CMK-3 + 10, 20, 30, 40, or 50 mg of PSA).

The effect of CMK-3 dosage on the overall pesticide recoveries were investigated in the range of 5–20 mg. The extraction solution (1 mL) was added to the centrifuge tube containing 150 mg of anhydrous MgSO4 and different amounts of CMK-3 (5, 7.5, 10, 15, or 20 mg). The recovery distributions of the pesticides are shown in Fig. 2A. It was evident that the percentage of pesticides with recoveries less than 70% increased with increasing CMK-3 dosage. In particular, when the amount of CMK-3 exceeded 7.5 mg, the recoveries of more than 14.9% of pesticides were below 70%. This result demonstrated that some pesticides might be adsorbed at high amounts of CMK-3 (10, 15, or 20 mg). When 5 mg or 7.5 mg CMK-3 was used, there were no remarkable differences in the percentages of pesticides with recoveries in the range of 70–120%, both were more than 93.9%. However, 65.8% of the pesticide recoveries fell into the more satisfactory range of 90–110% with 5 mg of CMK-3, and only 50.0% using 7.5 mg of CMK-3. Therefore, 5 mg of CMK-3 was chosen as the optimal amount and used in the following experiments.

#### 3.2.2. Dosage of PSA

The effect of different PSA dosages on pesticide recoveries from *Fritillaria* samples were investigated by adding 150 mg of anhydrous MgSO_4_, 5 mg of CMK-3, and different amounts of PSA (10, 20, 30, 40, or 50 mg) to 1 mL of the extraction solution. After purification, the supernatant was analyzed by LC-MS/MS to calculate the recoveries of each pesticide. As shown in Fig. 2B, as the amount of PSA increased from 10 mg to 30 mg, the percentage of pesticide with recoveries in the range of 90–110% increased from 57.9% to 72.8%. However, the percentage of pesticides with recoveries in the range of 90–110% decreased when the amount of PSA exceeded 30 mg. Thus, 30 mg was chosen as the optimized amount.

In summary, the modified QuEChERS method for the analysis of multi-pesticide residues in *Fritillaria* samples utilized 6 mL of ACN as the extraction solvent, and 5 mg of CMK-3 and 30 mg of PSA as the purification sorbents.

#### 3.2.3. Evaluation of purification effect

Matrix effects were known to occur frequently in pesticide residue analysis and may affect the accuracy of the analysis results. An effective purification process was needed to reduce matrix interference and improve robustness of the analytical method. However, two problems were noticed during the analysis of experimental data. First of all, the lowest dosage was selected due to the poor recovery of the high dosage in Section 3.2.1. So, does 5 mg of CMK-3 play a good role in removing impurities? Moreover, two pesticides with recoveries less than 70%, prochloraz (64.4%) and pyraclostrobin (67.5%), were found under the optimal conditions in Section 3.2.2. But there is no pesticide with recoveries less than 70% under the unpurified conditions in Section 3.1. Hence, considering these two issues, the purification effect of the selected sorbents was investigated to evaluate whether the established method has advantages over the non-purification. The matrix effects of pesticides in unpurified, CMK-3 (5 mg) purified and CMK-3 (5 mg) + PSA (30 mg) purified samples were compared to find differences. In this experiment, the matrix effect was calculated using the peak area ratio of the pesticides in the standard solutions at 20 μg L^−1^ [eqn (2)]. The blank matrix extraction solution without purification, or with two strategy purification was used to prepare the matrix-matched standard solution by spiking the target analytes at 20 μg L^−1^, respectively.

**Fig. 3:**
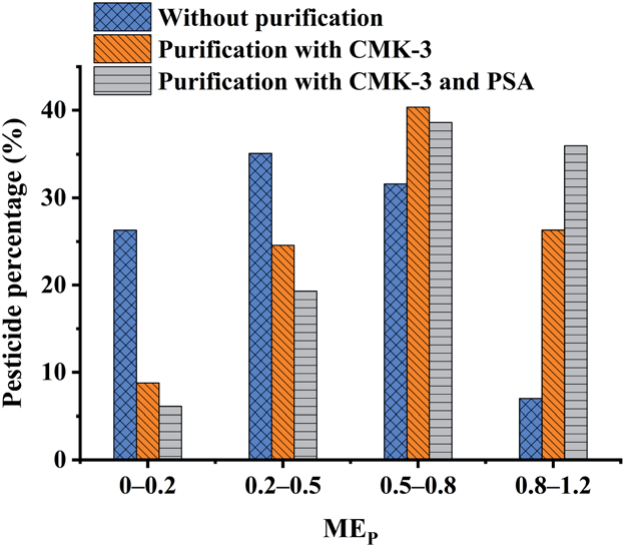
Comparison of the matrix effects between the unpurified, CMK-3 purified and CMK-3 (5 mg) + PSA (30 mg) purified matrix-matched solutions at 20 μg L^−1^.

**Fig. 4:**
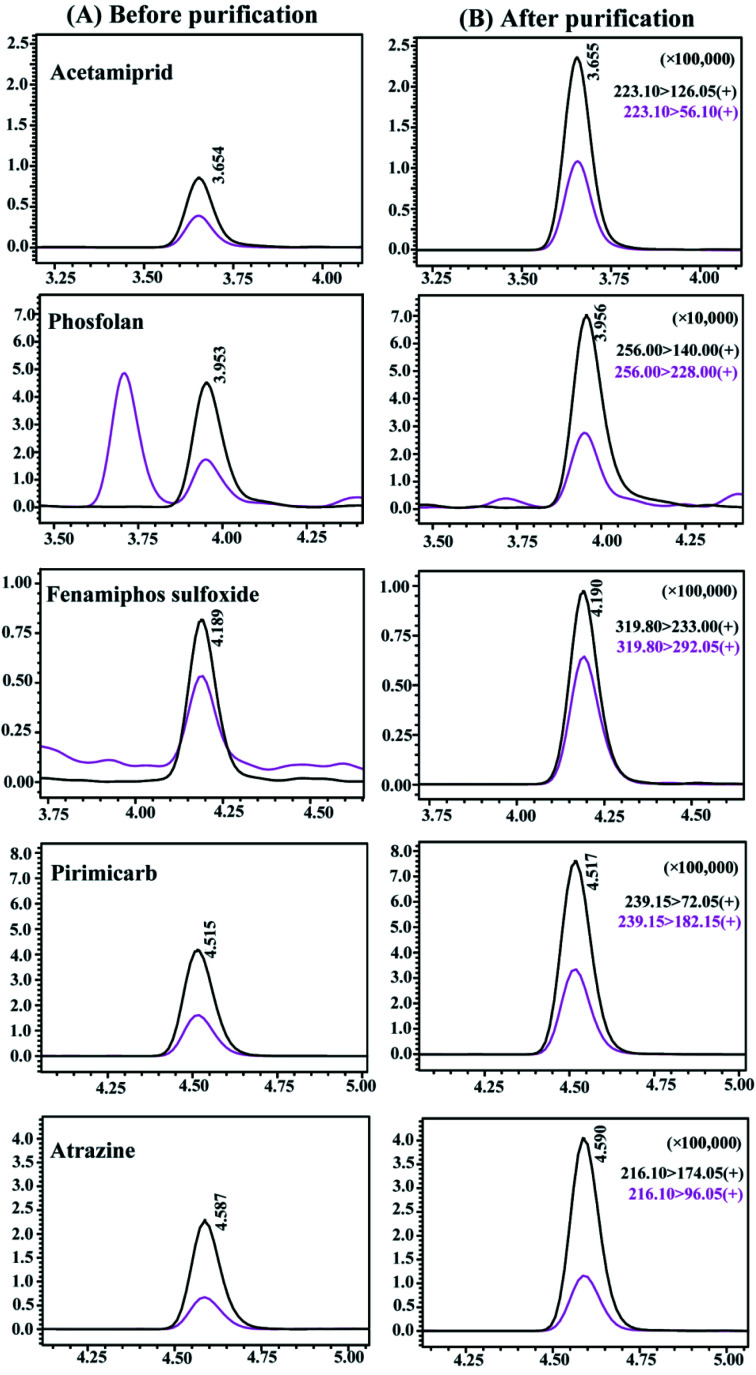
LC-MS/MS chromatograms of five representative pesticides before (A) and after (B) purification with CMK-3 and PSA. The spiked concentration was 20 μg L^−1^, and the retention time and ion pair data were given.

The ME_P_ values of all pesticides with different purification methods are summarized in Fig. 3. The ME_P_ values of all pesticides were classified into four groups according to the interference degree of the matrix compounds on the target analytes, where ME_P_ values of 0–0.2 indicated strong signal suppression, 0.2–0.5 indicated relatively lower signal suppression than those in the range of 0–0.2, 0.5–0.8 indicated medium signal suppression, and 0.8–1.2 indicated low signal suppression or enhancement. Comparing with the unpurified treatment, the percentage of pesticides with ME_P_ values in the range of 0–0.5 decreased from 61.4% to 33.3%, and the percentage of pesticides with ME_P_ of 0.5–1.2 increased from 38.6% to 66.7% by using CMK-3. In particular, the percentages of pesticides within the range of 0–0.2 and 0.8–1.2 were significantly changed, with decrease of 17.5% of pesticides and increase of 19.3% of pesticides, respectively. It is confirmed that even 5 mg of CMK-3 made a significant contribution to improving matrix effects. PSA was additionally coupled with CMK-3 as purification sorbents to reduce the percentages of pesticides with ME_P_ values in the range of 0–0.8, and again increased by 9.65% of pesticides in the range of 0.8–1.2. Compared to the unpurified treatment, the optimal purification treatment (CMK-3 + PSA) gave rise to an increase of the percentage of pesticides with ME_P_ of 0.8–1.2 from 7.02% to 36.0%. It can be concluded that the use of CMK-3 and PSA played a good cleanup role in the purification process of *Fritillaria* matrix. In addition, the chromatographic peaks of pesticides under different treatments were compared and the chromatograms of some representative pesticides are presented in Fig. 4. It can be clearly observed that the responses of some pesticides were enhanced and the impurity peaks of the sample matrix were reduced after purification. The above results indicated that the combination of CMK-3 and PSA effectively reduced the interference of the *Fritillaria* matrix components on the target analytes such that the quantitative results of the target analytes were closer to the actual levels in a pure solvent. Moreover, the purified sample matrix could reduce the contamination of the instrument and protect the instrument. Thus, it was acceptable to effectively improve the matrix effect of samples and obtain less quantitative error at the cost of a slight decrease in the recoveries of a few pesticides. Furthermore, the matrix effects of the purified extracts obtained by the peak area ratio of the standard solution in this experiment were similar to those of the FT sample obtained by the slope ratio of the method validation calibration curve.

### 3.3. Method validation

The applicability of the developed method was validated in all *Fritillaria* samples (FT, FU, FC and FD) by evaluating its linearity, matrix effect, recovery, precision, LOD and LOQ.

#### 3.3.1. Linearity

For linearity and linear range, the calibration curves were fitted to linear regression equations and the results are presented in Tables S2–S5.† Good linearity results with *R* ranging from 0.9947 to 0.9999 were observed for all the pesticides in the four *Fritillaria* samples. However, the linear range of some pesticides varied because of the different responses of each pesticide and interferences from the matrix effects of different *Fritillaria* samples. There were 79.8% of the pesticides in the linear range of 1–250 μg L^−1^ and 13.2% of the pesticides between 1–100 μg L^−1^ for all of the four *Fritillaria* samples. The linearity of 3-hydroxy-carbofuran, triadimefon, terbufos, fenpropathrin, mevinphos, phorate sulfone, methoxyfenozide and cyanofenphos ranged from 2 μg L^−1^ to 250 μg L^−1^ in some *Fritillaria* samples owing to their relatively lower responses at 1 μg L^−1^.

#### 3.3.2. Matrix effect

The matrix effects of the four *Fritillaria* samples were evaluated according to eqn (1). The ME_S_ values of each pesticide from the four *Fritillaria* matrices are presented in Tables S2–S5.† The ME_S_ values of all pesticides in the four *Fritillaria* samples ranged from 0.06 to 1.25. The influence of various *Fritillaria* matrices on each target analyte was evaluated by classifying the ME_S_ values as follows: ME_S_ values of 0–0.5 are considered as severe matrix suppression, 0.5–0.8 are considered as medium matrix suppression, and 0.8–1.2 are commonly considered as weak matrix effects of signal suppression or enhancement and are acceptable. From Tables S2–S5,† it was observed that 4.39–8.77% of the pesticides displayed ME_S_ values in the range of 0–0.5 for the FU, FC, and FD samples, whereas more pesticides (24.6%) did the same for the FT sample. ME_S_ values of 18.4–37.7% of the pesticides were between 0.5–0.8 in all of the *Fritillaria* samples. ME_S_ values of 0.8–1.2 were observed for 36.8%, 76.3%, 71.9%, and 69.3% of pesticides in the FT, FU, FC, and FD samples, respectively. The above results indicated that the quantitative analysis of pesticide residues would be affected by the *Fritillaria* matrix from different species, therefore, it was necessary to use matrix-matched standard solutions to reduce quantitative errors.

#### 3.3.3. Recovery and precision

For each *Fritillaria* sample, the percentages of pesticides with recoveries between 70–120% at the spiked concentrations of 30, 120, and 240 μg kg^−1^ are displayed in Table 1. It was observed that 84.2–100% of the pesticide had satisfactory recovery results for all of the *Fritillaria* samples at the three spiked concentrations. However, it was found that the recoveries of several pesticides in FU, FC and FD samples were lower than 60% at the three spiked levels. Considering that different matrix components can affect the recovery of pesticides, this method was considered unsuitable for the detection of these pesticides in individual *Fritillaria*. Namely, the developed method was not suitable for detecting ethofenprox in FU, lufenuron and tolfenpyrad in FU and FC, carbendazim in FC and FD, and coumaphos, emamectin benzoate, prochloraz and pyraclostrobin in FU, FC and FD. Table 1 demonstrates the mean recoveries and corresponding RSDs of all pesticides for each *Fritillaria* sample at the three spiked concentrations. It was observed that the mean recoveries for all of the spiked levels were in the range of 88.6–95.5% with RSDs values of 3–6%. The overall mean recoveries across all of the spiked concentrations for the FT, FU, FC, and FD samples were 92.6%, 92.5%, 91.2%, and 90.9%, respectively, and the mean RSDs of all samples were below 5%. The above results show that the recovery and precision of the developed method fulfill the analytical criteria. All pesticide recoveries and RSD values for the *Fritillaria* samples have been summarized in Tables S6–S9.†

**Table 1:** Recoveries of the pesticides at spiked concentrations of 30, 120, and 240 μg kg^−1^ in the various *Fritillaria* samples

Samples	Mean recoveries (%) ± mean RSD (%) of pesticides at 30, 120 and 240 μg kg^−1^ in each *Fritillaria* sample				The percentages of pesticides with recoveries between 70–120% at 30, 120 and 240 μg kg^−1^ in each *Fritillaria* sample		
	30 μg kg^−1^	120 μg kg^−1^	240 μg kg^−1^	Mean ± RSD	30 μg kg^−1^ (%)	120 μg kg^−1^ (%)	240 μg kg^−1^ (%)
FT	94.1 ± 6	94.2 ± 4	89.4 ± 4	92.6 ± 4	98.2	100	98.2
FU	88.6 ± 5	95.5 ± 3	93.2 ± 4	92.5 ± 4	84.2	89.5	89.5
FC	89.3 ± 5	90.9 ± 4	93.4 ± 4	91.2 ± 4	86.0	91.2	93.0
FD	94.8 ± 6	89.0 ± 3	88.8 ± 4	90.9 ± 4	92.1	90.4	91.2
Minimum	88.6 ± 5	89.0 ± 3	88.8 ± 4				
Maximum	94.8 ± 6	95.5 ± 3	93.4 ± 4				

#### 3.3.4. LOD and LOQ

The sensitivity of the developed method was evaluated on the basis of instrument LODs and method LOQs. As demonstrated in Tables S2–S5,† the instrument LODs of all pesticides were in the range of 0.03–3.31 μg L^−1^ depending on the samples. The data in Tables S6–S9† reveals that the recoveries of all the pesticides were in the range of 60.9–118.7% with RSD less than 20% at the lowest spiked concentration of 30 μg kg^−1^, except for avermectin and fipronil. Thus, the method LOQs of all the pesticides were 30 μg kg^−1^, except that for avermectin (120 μg kg^−1^) in the FU samples and fipronil (120 μg kg^−1^) in the FU and FC samples.

Overall, these validation results indicate that the developed method for multi-residue analysis of pesticides has satisfactory linearity, matrix effect, recovery, precision and sensitivity, and is suitable for the detection of pesticide residues in various *Fritillaria* species.

### 3.4. Application to real samples

Forty-seven *F. thunbergii* samples from Zhejiang province, China, were analyzed using the optimized method. The analysis revealed that seven pesticides were present in twenty-two samples in contaminated concentrations of 0.030–0.203 mg kg^−1^. Among all the detected samples, seven samples contained two pesticides, and one sample contained four pesticides. The sample analysis results were evaluated based on the MRL of other herbal medicines, such as ginseng, set by China and the EU, owing to the lack of MRLs of *Fritillaria*. Details of the detected pesticides and the corresponding MRLs are presented in Table 2. It was found that the detected frequency of carbendazim was 38.3%, which was the highest among all the detected pesticides, followed by fluopyram (10.6%), difenoconazole (6.38%), thiophanate-methyl (4.26%), dimethomorph (4.26%), tebuconazole (2.13%), and propiconazole (2.13%). Referring to the MRL regulations of other herbs issued by China and the EU, it was observed that the residue levels of detected carbendazim in two samples exceeded the EU MRLs (Fig. S1†), but none of the detected pesticides exceeded the Chinese MRLs. Multiple pesticides were detected simultaneously in *Fritillaria* samples, which revealed the severity of pesticide abuse in the cultivation of *Fritillaria*, confirming the necessity and urgency for monitoring the multi-pesticide residues in the quality and safety supervision of *Fritillaria* products.

**Table 2:** The frequencies and concentration ranges of the detected pesticides in real *Fritillaria* samples and the number of samples exceeding MRL

Detected pesticides	CN MRL^a^ (mg kg^−1^)	EU MRL (mg kg^−1^)	Frequency and concentration range of detected pesticides		No.
			Frequency (%)	Range (mg kg^−1^)	
Carbendazim	1.00	0.10	38.3	0.030–0.203	2
Thiophanate-methyl	—b	0.10	4.26	0.034, 0.094	
Dimethomorph	—	0.05	4.26	0.034, 0.042	
Fluopyram	—	2.50	10.6	0.036–0.084	
Tebuconazole	3.00	0.15	2.13	0.048	
Propiconazole	0.10	0.05	2.13	0.037	
Difenoconazole	0.50	20.00	6.38	0.034–0.057	

a The “CN MRL” represents the “Chinese MRL”.

b The “—” represents the MRL for herbs is not set by China.

## 4. Conclusions

In this study, a modified QuEChERS coupled with LC-MS/MS method was developed for the simultaneous determination of 114 pesticides in *Fritillaria*. In view of the complexity of *Fritillaria* matrix and abundant medicinal species, the process of the QuEChERS method was systematically optimized, and the combination of CMK-3 and PSA was selected to effectively reduce the matrix effect. To the best of our knowledge, the present work is the first to apply CMK-3 as cleanup sorbent in pesticide residue analysis to remove matrix interference in *Fritillaria*. The established method was fully validated in four *Fritillaria* samples, and good linearity, weak matrix effect, satisfactory recovery, good precision and high sensitivity were obtained, demonstrating its applicability for the analysis of various *Fritillaria* species. Moreover, the method was successfully applied to 47 real *Fritillaria* samples from Zhejiang province, China, of which 22 samples were found to have residues of one or more pesticides. Among all of the seven detected pesticides, carbendazim exceeded EU MRLs in two samples but did not exceed the Chinese MRLs. These results demonstrated that this method is suitable for the analysis of multiple pesticide residues in *Fritillaria* and can be used as a helpful tool in routine monitoring of the quality of medicinal *Fritillaria*.

## Conflicts of interest

There are no conflicts to declare.

## Supplementary Material

RA-011-D0RA07229J-s001
