# Supplementary material for: Simultaneous determination of 114 pesticides in complex Chinese herbal medicine Fritillaria using ordered mesoporous carbon CMK-3 as a reversed-dispersive solid phase extraction sorbent
Source: RSC Adv. 2021 Jan 20;11(7):4129–37. doi: 10.1039/d0ra07229j (PMC8694350; doi:10.1039/d0ra07229j)
Supplement: RA-011-D0RA07229J-s001 [file RA-011-D0RA07229J-s001.pdf]

Electronic Supplementary Material (ESI) for RSC Advances.
This journal is © The Royal Society of Chemistry 2021

Supplementary materials

Simultaneous determination of 114 pesticides in complex Chinese herbal medicine *Fritillaria* using ordered mesoporous carbon CMK-3 as a reversed-dispersive solid phase extraction sorbent

Tong Wu[a], Peipei Qi[b,c,d,1], Jiao Wang[b,c,d], Zhiwei Wang[b,c,d], Shanshan Di[b,c,d], Hao Xu[b,c,d], Huiyu Zhao[b,c,d], Changshan Zhao[a,*], Xinquan Wang[b,c,d,*]

[a] College of Agriculture, Northeast Agricultural University, Harbin, 150030, P. R. China

[b] State Key Laboratory for Managing Biotic and Chemical Threats to the Quality and Safety of Agro-products, Institute of Quality and Standard of Agro-products, Zhejiang Academy of Agricultural Sciences, Hangzhou 310021, P. R. China

[c] Agricultural Ministry Key Laboratory for Pesticide Residue Detection, Hangzhou 310021, P. R. China

[d] Key Laboratory of Detection for Pesticide Residue and Control of Zhejiang, Hangzhou 310021, P. R. China

* Corresponding author:

Professor Xinquan Wang

Postal address: No. 198 Shiqiao Road, Hangzhou 310021, P. R. China

Tel: + 86 571 86419051; E-mail address: wangxinquan212@163.com

Professor Changshan Zhao

Postal address: No. 600 Changjiang Road, Harbin 150030, P. R. China

Tel: + 86451 55191775; Email address: csz-hlj@sohu.com

[1] Peipei Qi is the co-first author owing to the equal contribution.

Table S1 Parameters for 114 pesticides analysis by LC-MS/MS.

Pesticides	Parent ion (m/z)	Product ion (m/z)	Q1 prerod (V)	Collision energy (eV)	Q3 prerod (V)
Acephate	184.20	143.00;95.00	-20;-20	-8;-23	-15;-16
Acetamiprid	223.10	126.05;56.10	-30;-30	-22;-15	-30;-23
Alachlor	270.10	238.05;162.15	-30;-30	-10;-19	-26;-30
Atrazine	216.10	174.05;96.05	-30;-30	-17;-25	-18;-17
Avermectin	895.50	449.30;751.50	-32;-32	-46;-46	-32;-22
Azaconazole	300.00	159.00;231.00	-15;-15	-27;-17	-29;-23
Azamethiphos	325.00	183.10;112.10	-16;-16	-16;-38	-19;-20
Azoxystrobin	404.10	372.05;329.00	-30;-30	-14;-31	-26;-23
Benalaxyl	326.20	148.20;294.10	-16;-16	-21;-11	-15;-20
Bendiocarb	224.10	167.05;109.05	-25;-25	-9;-19	-18;-20
Bitertanol	338.20	269.15;99.10	-17;-17	-9;-15	-29;-18
Bromuconazole	377.90	158.90;70.00	-19;-19	-28;-23	-30;-30
Buprofezin	306.10	201.10;116.10	-30;-30	-11;-16	-22;-12
Cadusafos	271.10	159.00;97.00	-30;-30	-14;-37	-29;-18
Carbendazim	192.05	160.05;132.05	-30;-30	-17;-30	-30;-24
Carbofuran	222.10	165.10;123.10	-25;-25	-11;-21	-17;-22
Chlorantraniliprole	484.00	452.90;285.85	-24;-24	-19;-16	-30;-30
Chlorpyrifos	351.90	199.90;96.95	-27;-27	-18;-33	-21;-18
Chlortoluron	213.10	72.00;140.10	-23;-24	-21;-23	-28;-24
Clothianidin	250.00	169.10;132.00	-29;-29	-12;-14	-17;-24

Coumaphos	363.00	227.00;307.10	-18;-18	-26;-18	-23;-21
Cyanofenphos	304.10	77.25;122.20	-20;-11	-60;-19	-16;-25
Cyazofamid	325.00	108.05;261.10	-23;-23	-12;-10	-21;-28
Cyproconazole	292.10	70.05;125.05	-30;-30	-20;-30	-27;-22
Dicrotophos	238.00	112.10;193.00	-12;-12	-12;-9	-11;-20
Difenoconazole	406.10	251.00;337.05	-30;-30	-25;-17	-27;-24
Dimethoate	230.00	198.95;125.00	-26;-26	-9;-22	-21;-22
Dimethomorph	388.10	301.00;165.05	-19;-19	-20;-34	-21;-30
Dimethyl phthalate	195.10	163.30;77.25	-13;-13	-10;-31	-19;-15
Diniconazole	326.10	70.00;159.00	-16;-16	-25;-30	-28;-30
Dinotefuran	203.10	129.10;113.10	-22;-22	-12;-10	-22;-12
Emamectin Benzoate	886.40	158.30;82.25	-26;-26	-40;-70	-12;-17
Epoxiconazol	330.10	121.20;141.10	-17;-17	-21;-18	-22;-25
Ethiofencarb	226.10	107.05;164.05	-26;-26	-15;-8	-19;-30
Ethofenprox	394.00	177.10;106.95	-19;-19	-17;-40	-20;-19
Etoxazole	360.00	141.00;113.00	-15;-15	-30;-35	-15;-15
Fenamiphos	304.10	217.10;202.00	-15;-15	-22;-36	-23;-21
Fenamiphos sulfoxide	319.80	233.00;292.05	-30;-30	-23;-16	-26;-21
Fenbuconazole	336.90	125.05;70.00	-26;-26	-27;-20	-25;-28
Fenpropathrin	350.30	125.20;97.20	-24;-25	-18;-31	-12;-17
Fipronil	435.00	330.00;250.00	10;10	16;28	21;24
Fipronil-sulfone	450.90	415.00;282.25	22;22	16;27	10;12
Fludioxonil	247.10	180.20;126.15	17;17	29;32	30;22

Fluopyram	397.10	173.10;208.10	-28;-15	-28;-22	-24;-21
Fluoxastrobin	458.90	427.20;188.30	-17;-22	-20;-36	-17;-14
Flusilazole	316.10	247.10;165.10	-30;-30	-18;-29	-27;-30
Flutolanil	324.10	262.10;242.00	-16;-16	-18;-26	-27;-25
Flutriafol	302.10	123.00;109.00	-15;-15	-28;-31	-22;-19
Hexaconazole	314.10	70.20;159.15	-15;-15	-21;-29	-28;-30
Hexaflumuron	459.00	438.90;175.10	16;16	12;36	29;29
Hexazinone	253.15	171.10;85.10	-30;-30	-15;-31	-18;-15
3-Hydroxy-carbofuran	238.10	163.10;181.20	-27;-27	-14;-10	-17;-19
Imazalil	297.00	159.00;200.00	-15;-15	-23;-19	-15;-15
Imidacloprid	256.05	175.10;209.05	-29;-29	-17;-14	-18;-22
Indoxacarb	528.10	249.10;292.95	-26;-26	-17;-15	-27;-21
Ipconazole	334.00	70.30;125.20	-23;-23	-23;-47	-14;-14
Isoprocarb	194.10	95.00;137.10	-21;-22	-14;-10	-17;-14
Kresoxim-methyl	314.00	222.30;267.30	-15;-21	-17;-8	-12;-14
Lufenuron	509.00	326.00;339.00	24;24	19;12	15;22
Machette	312.20	238.10;147.20	-15;-15	-12;-36	-26;-28
Malaoxon	314.90	98.95;127.00	-15;-15	-24;-13	-19;-23
Malathion	331.00	127.05;99.00	-17;-17	-12;-23	-13;-18
Mepronil	270.15	119.05;228.05	-30;-30	-25;-18	-30;-30
Metalaxyl	280.10	220.20;192.20	-30;-30	-13;-18	-24;-20
Metalaxyl-M	280.10	220.15;248.10	-30;-30	-13;-10	-24;-27
Methidathion	303.00	145.00;85.10	-21;-21	-8;-22	-15;-30

Methomyl	163.05	88.00;106.05	-18;-18	-8;-10	-16;-19
Methoprene	311.10	117.30;119.15	-22;-15	-52;-31	-14;-22
Methoxyfenozide	369.20	149.10;313.10	-18;-18	-16;-8	-16;-22
Mevinphos	225.00	127.05;193.05	-25;-25	-17;-8	-23;-20
Monocrotophos	224.10	127.05;193.00	-25;-25	-15;-8	-13;-20
Myclobutanil	289.10	70.05;125.05	-30;-30	-21;-30	-28;-22
Omethoate	214.10	183.00;155.00	-23;-23	-10;-14	-19;-28
Paclobutrazol	294.10	70.05;125.05	-15;-15	-21;-40	-28;-22
Penconazole	284.10	159.00;70.00	-14;-14	-27;-17	-30;-27
Phenthoate	321.00	79.10;246.95	-23;-23	-41;-11	-30;-17
Phorate	261.00	75.00;143.00	-29;-29	-10;-18	-30;-15
Phorate sulfone	293.00	171.05;115.00	-22;-22	-9;-24	-19;-20
Phosalone	368.00	182.05;111.00	-30;-30	-14;-39	-19;-20
Phosfolan	256.00	140.00;228.00	-13;-13	-24;-12	-26;-25
Phosphamidon	300.00	174.05;127.00	-15;-15	-12;-29	-17;-22
Piperonyl butoxide	356.30	177.10;119.00	-24;-24	-13;-37	-19;-22
Pirimicarb	239.15	72.05;182.15	-30;-30	-25;-19	-30;-30
Pirimiphos-methyl	306.05	108.10;95.00	-30;-30	-31;-29	-19;-17
Prochloraz	376.00	308.00;266.00	-19;-19	-11;-17	-21;-29
Profenofos	372.90	302.80;345.00	-18;-18	-19;-12	-30;-24
Propamocarb	189.20	102.05;144.05	-30;-30	-20;-12	-23;-15
Propiconazole	342.05	159.10;205.10	-17;-17	-30;-18	-29;-21
Pyraclostrobin	388.00	163.30;164.35	-11;-19	-24;-18	-18;-12

Pyridaben	365.10	309.05;147.10	-18;-18	-12;-25	-22;-27
Pyridafenthion	341.10	189.10;205.10	-17;-23	-22;-22	-20;-22
Pyrimitate	334.10	198.05;182.10	-25;-25	-22;-22	-21;-19
Pyrisoxazole	289.00	151.00;120.00	-15;-15	-20;-28	-15;-15
Simeconazole	294.10	70.05;135.05	-15;-15	-21;-21	-28;-24
Spirodiclofen	411.10	71.20;313.05	-21;-21	-16;-11	-28;-22
Sulfotep	323.00	115.00;171.10	-16;-16	-31;-15	-20;-18
Sulprofos	322.90	219.05;139.25	-16;-16	-16;-30	-16;-15
Tebuconazole	308.10	70.10;125.00	-22;-22	-22;-38	-27;-23
Tebufenozide	353.20	133.10;297.10	-18;-18	-20;-8	-24;-15
Tebufenpyrad	334.20	117.05;145.00	-16;-16	-38;-27	-21;-26
Temephos	467.00	419.00;341.00	-23;-23	-19;-32	-30;-24
Terbufos	289.00	103.15;57.10	-14;-14	-9;-24	-18;-24
Tetrachlorvinphos	364.90	127.05;203.90	-27;-27	-14;-38	-13;-21
Tetraconazole	372.00	159.05;70.20	-27;-27	-31;-24	-29;-27
Thiacloprid	253.00	126.05;99.00	-28;-28	-20;-43	-22;-17
Thiamethoxam	292.00	211.10;181.10	-30;-30	-11;-23	-22;-19
Thiophanate-methyl	343.05	151.15;311.05	-17;-17	-20;-10	-16;-22
Tolfenpyrad	384.10	197.10;154.05	-10;-10	-25;-41	-12;-29
Triadimefon	294.10	69.15;197.05	-21;-21	-22;-15	-26;-21
Triadimenol	296.10	70.05;99.15	-15;-15	-11;-15	-29;-17
Trifloxystrobin	409.10	186.05;145.00	-20;-20	-18;-44	-20;-26
Triticonazole	318.10	70.05;125.05	-16;-16	-21;-39	-28;-23

Uniconazole	292.10	69.84;124.85	-15;-15	-35;-35	-15;-15
Vitavax	236.05	143.00;124.00	-27;-27	-14;-20	-15;-22

Table S2 The linearity, LOD and ME of each pesticide in FT.

Pesticides	[1]y=a x+b	R	Linear range, μg L^{-1}	LOD, μg L^{-1}	ME
Acephate	[2]y =67444.2 x + 165489	0.9995	1-250	0.11	0.71
	[3]y = 95454.7 x + 126007	0.9998	1-250		
Acetamiprid	y = 59014.0 x + 183931	0.9990	1-250	0.06	0.27
	y = 216310 x + 859034	0.9996	1-250		
Alachlor	y = 1158360 x + 2973800	0.9974	1-100	0.90	0.88
	y = 1319150 x + 3664510	0.9956	1-100		
Atrazine	y = 71845.0 x + 333514	0.9989	1-250	0.45	0.44
	y = 163204 x + 1200030	0.9981	1-250		
Avermectin	y = 12432.4 x + 16595.4	0.9986	1-250	1.39	0.79
	y = 15804.3 x + 78893.4	0.9978	1-250		
Azaconazole	y = 165130 x + 593435	0.9996	1-250	0.73	0.63
	y = 264001 x + 1277940	0.9993	1-250		
Azamethiphos	y = 21863.4 x + 52802.6	0.9993	1-250	0.54	0.25
	y = 88694.2 x - 180974	0.9985	1-250		
Azoxystrobin	y = 513660 x + 1022300	0.9995	1-100	1.43	0.68
	y = 758953 x + 1340200	0.9989	1-100		
Benalaxyl	y = 127414 x + 890519	0.9972	1-250	0.32	0.85
	y = 149996 x + 734635	0.9988	1-250		
Bendiocarb	y = 35610.4 x + 145697	0.9978	1-250	0.72	0.36
	y = 98646.2 x + 217493	0.9996	1-250		
Bitertanol	y = 10484.9 x + 68699.1	0.9989	1-250	1.42	0.96
	y = 10941.0 x + 27922.0	0.9991	1-250		
Bromuconazole	y = 24847.2 x + 86690.0	0.9990	1-250	3.31	0.70
	y = 35549.1 x + 205283	0.9991	1-250		
Buprofezin	y = 297695 x + 2088270	0.9959	1-250	0.04	1.01

	y = 296179 x + 1739250	0.9981	1-250		
Cadusafos	y = 219799 x + 882700	0.9987	1-250	0.83	0.72
	y = 305975 x + 1489500	0.9990	1-250		
Carbendazim	y = 28169.9 x + 1723470	0.9947	1-250	0.35	0.09
	y = 320186 x + 1825120	0.9988	1-250		
Carbofuran	y = 76307.5 x + 291203	0.9991	1-250	0.21	0.24
	y = 315291 x + 1980730	0.9990	1-250		
Chlorantraniliprole	y = 33455.9 x + 206559	0.9977	1-250	0.59	0.55
	y = 60806.5 x + 374502	0.9984	1-250		
Chlorpyrifos	y = 1120950 x + 1247400	0.9997	1-100	0.17	0.96
	y = 1170180 x + 1250410	0.9991	1-100		
Chlortoluron	y = 44027.5 x + 78278.2	0.9994	1-250	0.64	0.20
	y = 224329 x + 1248420	0.9994	1-250		
Clothianidin	y = 28034.2 x + 80933.9	0.9995	1-250	0.15	0.42
	y = 66466.1 x + 259289	0.9995	1-250		
Coumaphos	y = 173475 x + 1174690	0.9972	1-250	0.13	0.92
	y = 187969 x + 1204260	0.9983	1-250		
Cyanofenphos	y = 33068.0 x + 177828	0.9992	2-250	1.35	0.88
	y = 37552.0 x + 261111	0.9988	2-250		
Cyazofamid	y = 39266.4 x + 302492	0.9955	1-250	0.62	0.47
	y = 84102.7 x + 400697	0.9995	1-250		
Cyproconazole	y = 131468 x + 472293	0.9995	1-250	0.12	0.81
	y = 161982 x + 841769	0.9989	1-250		
Dicrotophos	y = 40116.2 x + 110419	0.9996	1-250	0.19	0.70
	y = 57100.5 x + 80168.3	0.9999	1-250		
Difenoconazole	y = 145865 x + 727039	0.9993	1-250	0.09	0.99
	y = 147556 x + 675191	0.9984	1-250		
Dimethoate	y = 60545.5 x + 142407	0.9997	1-250	0.16	0.25

	y = 240641 x + 586878	0.9998	1-250		
Dimethomorph	y = 139257 x + 590544	0.9983	1-250	0.18	0.66
	y = 212219 x + 858828	0.9991	1-250		
Dimethyl phthalate	y = 18381.6 x + 535526	0.9993	1-250	0.63	0.15
	y = 125246 x + 2067560	0.9999	1-250		
Diniconazole	y = 46021.5 x + 161122	0.9994	1-250	0.43	0.84
	y = 54617.9 x + 134041	0.9997	1-250		
Dinotefuran	y = 47595.9 x + 188076	0.9993	1-250	0.10	0.75
	y = 63144.0 x + 193831	0.9997	1-250		
Emamectin Benzoate	y = 334745 x + 973657	0.9992	1-250	0.46	1.03
	y = 325660 x + 628673	0.9994	1-250		
Epoxiconazol	y = 86654.8 x + 722157	0.9991	1-250	0.33	0.76
	y = 114171 x + 775284	0.9975	1-250		
Ethiofencarb	y = 589194 x + 677383	0.9995	1-100	0.57	0.40
	y = 1478020 x + 2025200	0.9990	1-100		
Ethofenprox	y = 223592 x + 236175	0.9988	1-250	0.16	0.85
	y = 263965 x + 864543	0.9987	1-250		
Etoxazole	y = 759375 x + 2286160	0.9974	1-100	0.21	1.00
	y = 758833 x + 1867190	0.9985	1-100		
Fenamiphos	y = 309561 x + 2208160	0.9980	1-250	0.55	0.91
	y = 339535 x + 3259620	0.9975	1-250		
Fenamiphos sulfoxide	y = 68375.9 x + 157169	0.9993	1-250	0.48	0.51
	y = 133042 x + 550303	0.9994	1-250		
Fenbuconazole	y = 49225.7 x + 310779	0.9968	1-250	0.34	0.73
	y = 67466.4 x + 287280	0.9996	1-250		
Fenpropathrin	y = 8536.91 x + 74887.7	0.9971	2-250	1.08	0.85
	y = 10089.5 x + 53148.5	0.9988	2-250		
Fipronil	y = 8198.34 x + 25890.3	0.9983	1-250	1.21	0.75

	y = 10861.0 x + 47921.3	0.9971	1-250		
Fipronil-sulfone	y = 127209 x + 565983	0.9982	1-250	0.93	0.68
	y = 187287 x + 361756	0.9993	1-250		
Fludioxonil	y = 21209.7 x + 133422	0.9986	1-250	0.11	0.67
	y = 31485.4 x + 274109	0.9972	1-250		
Fluopyram	y = 125656 x + 438623	0.9992	1-250	0.15	0.66
	y = 189716 x + 1098190	0.9988	1-250		
Fluoxastrobin	y = 206791 x + 491519	0.9997	1-250	0.31	0.68
	y = 305857 x + 2315090	0.9976	1-250		
Flusilazole	y = 117996 x + 573059	0.9983	1-250	0.24	0.81
	y = 146458 x + 721624	0.9989	1-250		
Flutolanil	y = 191007 x + 904760	0.9991	1-250	0.43	0.57
	y = 333385 x + 2389380	0.9984	1-250		
Flutriafol	y = 16641.1 x + 46331.8	0.9991	1-250	0.21	0.40
	y = 41757.2 x + 226352	0.9991	1-250		
Hexaconazole	y = 58015.3 x + 246337	0.9993	1-250	0.14	0.90
	y = 64745.7 x + 399719	0.9983	1-250		
Hexaflumuron	y = 280357 x + 796514	0.9994	1-250	0.18	0.83
	y = 337835 x + 2789150	0.9972	1-250		
Hexazinone	y = 80015.8 x + 122487	0.9997	1-100	0.61	0.10
	y = 768340 x + 1387410	0.9996	1-100		
3-Hydroxy-carbofuran	y = 5869.19 x + 12461.5	0.9999	2-250	0.82	0.28
	y = 21261.4 x + 67880.3	0.9998	2-250		
Imazalil	y = 91053.5 x + 339121	0.9992	1-250	0.37	0.98
	y = 92868.5 x + 343302	0.9988	1-250		
Imidacloprid	y = 41506.1 x + 193546	0.9993	1-250	0.14	0.52
	y = 79673.5 x + 342956	0.9992	1-250		
Indoxacarb	y = 18408.4 x + 111598	0.9976	1-250	0.26	0.99

	y = 18648.3 x + 123693	0.9969	1-250		
Ipconazole	y = 158121 x + 313169	0.9995	1-250	0.18	0.89
	y = 178395 x + 768412	0.9985	1-250		
Isoprocarb	y = 45174.0 x + 133278	0.9994	1-250	0.23	0.36
	y = 126739 x + 933704	0.9986	1-250		
Kresoxim-methyl	y = 39041.8 x + 179831	0.9987	1-250	0.54	0.90
	y = 43423.6 x + 289101	0.9974	1-250		
Lufenuron	y = 10736.4 x + 119252	0.9966	1-250	1.01	0.92
	y = 11683.4 x + 152160	0.9943	1-250		
Machette	y = 193079 x + 1195940	0.9980	1-250	0.17	0.98
	y = 196230 x + 1188680	0.9978	1-250		
Malaoxon	y = 380149 x + 869837	0.9985	1-100	0.40	0.49
	y = 777428 x + 1903040	0.9992	1-100		
Malathion	y = 82921.6 x + 941505	0.9965	1-250	0.98	0.61
	y = 135705 x + 1538610	0.9979	1-250		
Mepronil	y = 1799300 x + 1250070	0.9994	1-100	0.36	0.47
	y = 3839890 x + 2734280	0.9993	1-100		
Metalaxyl	y = 291753 x + 502470	0.9992	1-100	0.65	0.37
	y = 797536 x + 1731510	0.9991	1-100		
Metalaxyl-M	y = 300943 x + 333923	0.9996	1-100	0.61	0.38
	y = 792866 x + 1973550	0.9990	1-100		
Methidathion	y = 22201.7 x + 111834	0.9988	1-250	0.51	0.67
	y = 33184.7 x + 229958	0.9964	1-250		
Methomyl	y = 51815.4 x + 193063	0.9993	1-250	0.08	0.72
	y = 71661.7 x + 193993	0.9996	1-250		
Methoprene	y = 25511.1 x + 92783.7	0.9988	1-250	0.51	0.94
	y = 27018.2 x + 138200	0.9980	1-250		
Methoxyfenozide	y = 52441.1 x + 212299	0.9993	1-250	0.58	0.72

	y = 72975.4 x + 455840	0.9984	1-250		
Mevinphos	y = 20202.1 x + 62303.5	0.9991	2-250	0.45	0.29
	y = 70676.6 x + 155188	0.9997	2-250		
Monocrotophos	y = 27251.2 x + 85852.1	0.9992	1-250	0.34	0.75
	y = 36326.6 x + 59398.2	0.9997	1-250		
Myclobutanil	y = 47880.6 x + 366437	0.9960	1-250	0.24	0.78
	y = 61274.2 x + 590971	0.9962	1-250		
Omethoate	y = 63881.7 x + 153465	0.9995	1-250	0.06	0.74
	y = 86878.8 x + 240660	0.9995	1-250		
Paclobutrazol	y = 243403 x + 1160070	0.9987	1-250	0.24	0.66
	y = 366191 x + 2096590	0.9988	1-250		
Penconazole	y = 72860.8 x + 630846	0.9959	1-250	0.11	0.79
	y = 92647.8 x + 648178	0.9979	1-250		
Phenthoate	y = 99214.8 x + 609397	0.9987	1-250	0.46	0.66
	y = 150919 x + 1088000	0.9984	1-250		
Phorate	y = 22784.8 x + 125411	0.9985	1-250	0.21	0.60
	y = 37916.5 x + 75618.0	0.9993	1-250		
Phorate sulfone	y = 13112.6 x + 21580.8	0.9995	1-250	0.98	0.33
	y = 39917.1 x + 287688	0.9986	1-250		
Phosalone	y = 1238670 x + 1236650	0.9995	1-100	1.35	0.93
	y = 1325190 x + 2320790	0.9984	1-100		
Phosfolan	y = 22325.7 x + 39560.9	0.9997	1-250	0.58	0.06
	y = 353707 x + 1927950	0.9993	1-250		
Phosphamidon	y = 12619.7 x + 20288.0	0.9994	1-250	0.51	0.09
	y = 143170 x + 102740	0.9998	1-250		
Piperonyl butoxide	y = 317689 x + 2380920	0.9957	1-250	0.26	1.01
	y = 313792 x + 1849940	0.9976	1-250		
Pirimicarb	y = 134737 x + 286822	0.9995	1-250	0.14	0.40

	y = 339671 x + 1637580	0.9994	1-250		
Pirimiphos-methyl	y = 257016 x + 895463	0.9983	1-250	0.19	0.80
	y = 321052 x + 1858120	0.9977	1-250		
Prochloraz	y = 124683 x + 609326	0.9984	1-250	0.09	0.81
	y = 153835 x + 1028100	0.9984	1-250		
Profenofos	y = 86940.2 x + 663632	0.9967	1-250	0.05	0.86
	y = 100949 x + 465548	0.9989	1-250		
Propamocarb	y = 258619 x + 191987	0.9996	1-250	0.10	1.25
	y = 206167 x - 219273	0.9996	1-250		
Propiconazole	y = 85740.8 x + 500916	0.9982	1-250	0.12	0.87
	y = 98529.3 x + 664585	0.9977	1-250		
Pyraclostrobin	y = 174712 x + 847122	0.9986	1-250	0.20	0.96
	y = 181948 x + 753832	0.9993	1-250		
Pyridaben	y = 323405 x + 1787080	0.9979	1-250	1.09	0.90
	y = 360922 x + 3184610	0.9919	1-250		
Pyridafenthion	y = 141796 x + 783650	0.9983	1-250	1.20	0.83
	y = 170224 x + 1467230	0.9976	1-250		
Pyrimitate	y = 1149080 x + 102967	0.9995	1-100	0.14	0.92
	y = 1245920 x + 367730	0.9997	1-100		
Pyrisoxazole	y = 19977.6 x + 81214.9	0.9996	1-250	0.15	0.77
	y = 26076.3 x + 108653	0.9987	1-250		
Simeconazole	y = 240805 x + 1175760	0.9986	1-250	0.13	0.68
	y = 353108 x + 2471750	0.9976	1-250		
Spirodiclofen	y = 41342.2 x + 132968	0.9992	1-250	0.23	0.94
	y = 43952.5 x + 194879	0.9984	1-250		
Sulfotep	y = 176366 x + 645895	0.9991	1-250	0.35	0.79
	y = 223151 x + 1500600	0.9968	1-250		
Sulprofos	y = 80558.1 x + 747512	0.9977	1-250	0.43	0.95

	y = 85161.1 x + 763653	0.9976	1-250		
Tebuconazole	y = 111005 x + 616399	0.9985	1-250	0.27	0.71
	y = 156376 x + 1092590	0.9961	1-250		
Tebufenozide	y = 43266.3 x + 142626	0.9995	1-250	1.91	0.70
	y = 61535.3 x + 320772	0.9979	1-250		
Tebufenpyrad	y = 93834.4 x + 603701	0.9974	1-250	0.16	1.01
	y = 92916.7 x + 528019	0.9965	1-250		
Temephos	y = 334720 x + 2484330	0.9983	1-250	0.07	0.97
	y = 343536 x + 2610770	0.9969	1-250		
Terbufos	y = 11341.7 x + 116840	0.9969	2-250	2.08	0.89
	y = 12725.3 x + 140710	0.9965	2-250		
Tetrachlorvinphos	y = 25855.5 x + 227391	0.9958	1-250	0.03	0.74
	y = 35145.0 x + 256671	0.9972	1-250		
Tetraconazole	y = 111531 x + 689719	0.9973	1-250	0.19	0.61
	y = 182889 x + 950755	0.9989	1-250		
Thiacloprid	y = 124932 x + 202867	0.9980	1-100	0.34	0.19
	y = 649638 x + 824747	0.9996	1-100		
Thiamethoxam	y = 82580.6 x + 349572	0.9989	1-250	0.05	0.62
	y = 133709 x + 386705	0.9998	1-250		
Thiophanate-methyl	y = 78690.0 x + 418767	0.9981	1-250	0.06	0.27
	y = 295481 x + 984774	0.9995	1-250		
Tolfenpyrad	y = 97148.5 x + 712050	0.9966	1-250	0.09	0.96
	y = 101463 x + 557008	0.9988	1-250		
Triadimefon	y = 38849.2 x + 274157	0.9982	2-250	1.43	0.83
	y = 46994.8 x + 450105	0.9987	2-250		
Triadimenol	y = 30426.2 x + 171105	0.9989	1-250	1.22	0.78
	y = 39055.2 x + 144867	0.9995	1-250		
Trifloxystrobin	y = 379508 x + 616692	0.9996	1-100	0.51	0.59

	y = 646293 x + 726873	0.9996	1-100		
Triticonazole	y = 70345.4 x + 167505	0.9996	1-250	0.44	0.71
	y = 99459.4 x + 431537	0.9986	1-250		
Uniconazole	y = 136936 x + 573920	0.9990	1-250	0.14	0.71
	y = 194158 x + 795576	0.9994	1-250		
Vitavax	y = 430037 x + 202035	0.9991	1-100	0.13	0.12
	y = 3487430 x + 2053310	0.9985	1-100		

[1] Calibration curves are expressed as regression lines (y = a x + b), where y is the peak area and x is the concentration (µg L^{-1}), a is the slope, b is the intercept and R is the correlation coefficient.

[2] The first calibration curve for each pesticide represents the matrix-matched calibration curve.

[3] The second calibration curve for each pesticide represents the solvent standard calibration curve.

Table S3 The linearity, LOD and ME of each pesticide in FU.

Pesticides	[1]y=a x+b	R	Linear range, μg L^{-1}	LOD, μg L^{-1}	ME
Acephate	[2]y = 83848.6 x + 36853.8	0.9999	1-250	0.28	0.88
	[3]y = 95454.7 x + 126007	0.9998	1-250		
Acetamiprid	y = 116746 x + 558228	0.9973	1-250	0.28	0.54
	y = 216310 x + 859034	0.9996	1-250		
Alachlor	y = 1313040 x + 2102990	0.9997	1-100	1.33	1.00
	y = 1319150 x + 3664510	0.9956	1-100		
Atrazine	y = 139855 x + 1059030	0.9964	1-250	0.24	0.86
	y = 163204 x + 1200030	0.9981	1-250		
Avermectin	y = 13242.1 x + 32802.4	0.9996	1-250	1.06	0.84
	y = 15804.3 x + 78893.4	0.9978	1-250		
Azaconazole	y = 244370 x + 1408030	0.9973	1-250	0.80	0.93
	y = 264001 x + 1277940	0.9993	1-250		
Azamethiphos	y = 82116.8 x + 202661	0.9991	1-250	0.23	0.93
	y = 88694.2 x - 180974	0.9985	1-250		
Azoxystrobin	y = 727885 x + 1611050	0.9961	1-100	1.00	0.96
	y = 758953 x + 1340200	0.9989	1-100		
Benalaxyl	y = 147812 x + 760647	0.9975	1-250	0.47	0.99
	y = 149996 x + 734635	0.9988	1-250		
Bendiocarb	y = 87598.0 x + 363280	0.9983	1-250	0.13	0.89
	y = 98646.2 x + 217493	0.9996	1-250		
Bitertanol	y = 10741.8 x + 58514.5	0.9992	1-250	1.71	0.98
	y = 10941.0 x + 27922.0	0.9991	1-250		
Bromuconazole	y = 32525.1 x + 180056	0.9970	1-250	1.78	0.91
	y = 35549.1 x + 205283	0.9991	1-250		
Buprofezin	y = 289338 x + 1562960	0.9984	1-250	0.03	0.98

	y = 296179 x + 1739250	0.9981	1-250		
Cadusafos	y = 190619 x + 495273	0.9997	1-250	1.53	0.62
	y = 305975 x + 1489500	0.9990	1-250		
Carbendazim	y = 108146 x + 1845410	0.9998	1-250	0.23	0.34
	y = 320186 x + 1825120	0.9988	1-250		
Carbofuran	y = 205492 x + 1011220	0.9982	1-250	0.29	0.65
	y = 315291 x + 1980730	0.9990	1-250		
Chlorantraniliprole	y = 62905.7 x + 344358	0.9975	1-250	0.51	1.03
	y = 60806.5 x + 374502	0.9984	1-250		
Chlorpyrifos	y = 1121380 x + 1464310	0.9993	1-100	0.12	0.96
	y = 1170180 x + 1250410	0.9991	1-100		
Chlortoluron	y = 163232 x + 1370070	0.9955	1-250	0.26	0.73
	y = 224329 x + 1248420	0.9994	1-250		
Clothianidin	y = 45353.5 x + 159716	0.9989	1-250	0.14	0.68
	y = 66466.1 x + 259289	0.9995	1-250		
Coumaphos	y = 201587 x + 1139780	0.9987	1-250	0.15	1.07
	y = 187969 x +1204260	0.9983	1-250		
Cyanofenphos	y = 35902.6 x + 168748	0.9987	1-250	0.42	0.94
	y = 38061.2 x + 194748	0.9990	1-250		
Cyazofamid	y = 56238.7 x + 187989	0.9996	1-250	0.43	0.67
	y = 84102.7 x + 400697	0.9995	1-250		
Cyproconazole	y = 149269 x + 367582	0.9997	1-250	0.08	0.92
	y = 161982 x + 841769	0.9989	1-250		
Dicrotophos	y = 56324.5 x + 132938	0.9997	1-250	0.33	0.99
	y = 57100.5 x + 80168.3	0.9999	1-250		
Difenoconazole	y = 100723 x + 228243	0.9998	1-250	0.13	0.68
	y = 147556 x + 675191	0.9984	1-250		
Dimethoate	y = 107520 x + 447380	0.9977	1-250	0.20	0.45

	y = 240641 x + 586878	0.9998	1-250		
Dimethomorph	y = 214509 x + 899166	0.9985	1-250	0.13	1.01
	y = 212219 x + 858828	0.9991	1-250		
Dimethyl phthalate	y = 102713 x + 1999330	0.9997	1-250	0.66	0.82
	y = 125246 x + 2067560	0.9999	1-250		
Diniconazole	y = 44907.8 x + 180906	0.9985	1-250	0.13	0.82
	y = 54617.9 x + 134041	0.9997	1-250		
Dinotefuran	y = 61236.4 x + 149237	0.9995	1-250	0.10	0.97
	y = 63144.0 x + 193831	0.9997	1-250		
Emamectin Benzoate	y = 336122 x + 1116430	0.9987	1-250	0.44	1.03
	y = 325660 x + 628673	0.9994	1-250		
Epoxiconazol	y = 105689 x + 2994140	0.9995	1-250	0.20	0.93
	y = 114171 x + 775284	0.9975	1-250		
Ethiofencarb	y = 1358710 x + 1128630	0.9996	1-100	0.48	0.92
	y = 1478020 x + 2025200	0.9990	1-100		
Ethofenprox	y = 293783 x + 242423	0.9986	1-250	0.10	1.11
	y = 263965 x + 864543	0.9987	1-250		
Etoxazole	y = 766552 x + 2063020	0.9980	1-100	0.17	1.01
	y = 758833 x + 1867190	0.9985	1-100		
Fenamiphos	y = 325516 x + 2852170	0.9955	1-250	0.94	0.96
	y = 339535 x + 3259620	0.9975	1-250		
Fenamiphos sulfoxide	y = 110182 x + 591528	0.9986	1-250	0.39	0.83
	y = 133042 x + 550303	0.9994	1-250		
Fenbuconazole	y = 60227.8 x + 401587	0.9973	1-250	0.72	0.89
	y = 67466.4 x + 287280	0.9996	1-250		
Fenpropathrin	y = 7367.21 x + 86720.4	0.9960	2-250	1.14	0.73
	y = 10089.5 x + 53148.5	0.9988	2-250		
Fipronil	y = 10780.8 x + 30409.4	0.9996	1-250	0.65	0.99

	y = 10861.0 x + 47921.3	0.9971	1-250		
Fipronil-sulfone	y = 161445 x + 548021	0.9984	1-250	0.34	0.86
	y = 187287 x + 361756	0.9993	1-250		
Fludioxonil	y = 31447.4 x + 281011	0.9977	1-250	0.08	1.00
	y = 31485.4 x + 274109	0.9972	1-250		
Fluopyram	y = 160962 x + 895861	0.9980	1-250	0.26	0.85
	y = 189716 x + 1098190	0.9988	1-250		
Fluoxastrobin	y = 282834 x + 1309120	0.9988	1-250	0.22	0.92
	y = 305857 x + 2315090	0.9976	1-250		
Flusilazole	y = 107310 x + 523591	0.9988	1-250	0.73	0.73
	y = 146458 x + 721624	0.9989	1-250		
Flutolanil	y = 322251 x + 2550380	0.9963	1-250	0.30	0.97
	y = 333385 x + 2389380	0.9984	1-250		
Flutriafol	y = 30707.9 x + 175757	0.9969	1-250	0.29	0.74
	y = 41757.2 x + 226352	0.9991	1-250		
Hexaconazole	y = 63430.1 x + 318117	0.9990	1-250	0.12	0.98
	y = 64745.7 x + 399719	0.9983	1-250		
Hexaflumuron	y = 313035 x + 2517720	0.9951	1-250	0.08	0.93
	y = 337835 x + 2789150	0.9972	1-250		
Hexazinone	y = 653882 x - 145414	0.9995	1-100	0.34	0.85
	y = 768340 x + 1387410	0.9996	1-100		
3-Hydroxy-carbofuran	y = 12646.7 x + 54763.2	0.9988	2-250	0.66	0.59
	y = 21261.4 x + 67880.3	0.9998	2-250		
Imazalil	y = 85996.6 x + 353852	0.9988	1-250	0.23	0.93
	y = 92868.5 x + 343302	0.9988	1-250		
Imidacloprid	y = 56668.0 x + 424640	0.9982	1-250	0.33	0.71
	y = 79673.5 x + 342956	0.9992	1-250		
Indoxacarb	y = 21694.2 x + 105009	0.9983	1-250	0.58	1.16

	y = 18648.3 x + 123693	0.9969	1-250		
Ipconazole	y = 171293 x + 635191	0.9989	1-250	0.12	0.96
	y = 178395 x + 768412	0.9985	1-250		
Isoprocarb	y = 110946 x + 735958	0.9974	1-250	0.19	0.88
	y = 126739 x + 933704	0.9986	1-250		
Kresoxim-methyl	y = 41565.4 x + 237216	0.9985	1-250	0.17	0.96
	y = 43423.6 x + 289101	0.9974	1-250		
Lufenuron	y = 12114.9 x + 134392	0.9956	1-250	1.23	1.04
	y = 11683.4 x + 152160	0.9943	1-250		
Machette	y = 202657 x + 1241520	0.9983	1-250	0.14	1.03
	y = 196230 x + 1188680	0.9978	1-250		
Malaoxon	y = 761406 x + 1286320	0.9990	1-100	0.46	0.98
	y = 777428 x + 1903040	0.9992	1-100		
Malathion	y = 113980 x + 1538680	0.9971	1-250	0.94	0.84
	y = 135705 x + 1538610	0.9979	1-250		
Mepronil	y = 3160010 x + 478991	0.9981	1-100	0.19	0.82
	y = 3839890 x + 2734280	0.9993	1-100		
Metalaxyl	y = 676746 x + 1490290	0.9979	1-100	0.55	0.85
	y = 797536 x + 1731510	0.9991	1-100		
Metalaxyl-M	y = 719594 x + 1522050	0.9984	1-100	0.53	0.91
	y = 792866 x + 1973550	0.9990	1-100		
Methidathion	y = 30541.0 x + 161431	0.9985	1-250	0.63	0.92
	y = 33184.7 x + 229958	0.9964	1-250		
Methomyl	y = 35435.6 x + 9213.96	0.9998	1-250	0.11	0.49
	y = 71661.7 x + 193993	0.9996	1-250		
Methoprene	y = 29030.0 x + 32855.7	0.9997	1-250	1.25	1.07
	y = 27018.2 x + 138200	0.9980	1-250		
Methoxyfenozide	y = 82322.0 x + 371476	0.9984	1-250	0.17	1.13

	y = 72975.4 x + 455840	0.9984	1-250		
Mevinphos	y = 47084.2 x + 111110	0.9996	1-250	0.58	0.66
	y = 70804.9 x + 132770	0.9997	1-250		
Monocrotophos	y = 26702.4 x + 11922.5	0.9999	1-250	0.46	0.74
	y = 36326.6 x + 59398.2	0.9997	1-250		
Myclobutanil	y = 59431.2 x + 592542	0.9965	1-250	0.38	0.97
	y = 61274.2 x + 590971	0.9962	1-250		
Omethoate	y = 88059.1 x + 151544	0.9998	1-250	0.27	1.01
	y = 86878.8 x + 240660	0.9995	1-250		
Paclobutrazol	y = 319684 x + 1763260	0.9972	1-250	0.28	0.87
	y = 366191 x + 2096590	0.9988	1-250		
Penconazole	y = 86244.6 x + 413768	0.9994	1-250	0.12	0.93
	y = 92647.8 x + 648178	0.9979	1-250		
Phenthoate	y = 118592 x + 775043	0.9985	1-250	0.09	0.79
	y = 150919 x + 1088000	0.9984	1-250		
Phorate	y = 22085.9 x + 111576	0.9984	1-250	0.30	0.58
	y = 37916.5 x + 75618.0	0.9993	1-250		
Phorate sulfone	y = 34169.8 x + 337596	0.9966	2-250	0.84	0.86
	y = 39625.3 x + 338644	0.9989	2-250		
Phosalone	y = 1295660 x + 2414800	0.9984	1-100	1.05	0.98
	y = 1325190 x + 2320790	0.9984	1-100		
Phosfolan	y = 196878 x + 381820	0.9997	1-250	0.26	0.56
	y = 353707 x + 1927950	0.9993	1-250		
Phosphamidon	y = 102921 x + 82522.9	0.9999	1-250	0.31	0.72
	y = 143170 x + 102740	0.9998	1-250		
Piperonyl butoxide	y = 319221 x + 1873990	0.9976	1-250	0.05	1.02
	y = 313792 x + 1849940	0.9976	1-250		
Pirimicarb	y = 325350 x + 1790040	0.9972	1-250	0.11	0.96

	y = 339671 x + 1637580	0.9994	1-250		
Pirimiphos-methyl	y = 252773 x + 950543	0.9988	1-250	0.07	0.79
	y = 321052 x + 1858120	0.9977	1-250		
Prochloraz	y = 149777 x + 638872	0.9990	1-250	0.06	0.97
	y = 153835 x + 1028100	0.9984	1-250		
Profenofos	y = 94934.9 x + 614688	0.9983	1-250	0.05	0.94
	y = 100949 x + 465548	0.9989	1-250		
Propamocarb	y = 241911 x + 87744.7	0.9999	1-250	0.17	1.17
	y = 206167 x - 219273	0.9996	1-250		
Propiconazole	y = 99753.9 x + 500845	0.9987	1-250	0.08	1.01
	y = 98529.3 x + 664585	0.9977	1-250		
Pyraclostrobin	y = 190042 x + 1186800	0.9986	1-250	0.15	1.04
	y = 181948 x + 753832	0.9993	1-250		
Pyridaben	y = 346464 x + 1918410	0.9985	1-250	0.53	0.96
	y = 360922 x + 3184610	0.9919	1-250		
Pyridafenthion	y = 185441 x + 1555610	0.9961	1-250	0.91	1.09
	y = 170224 x + 1467230	0.9976	1-250		
Pyrimitate	y = 1230110 x + 478425	0.9999	1-100	0.38	0.99
	y = 1245920 x + 367730	0.9997	1-100		
Pyrisoxazole	y = 22862.0 x + 9114.65	0.9998	1-250	0.40	0.88
	y = 26076.3 x + 108653	0.9987	1-250		
Simeconazole	y = 318647 x + 1666090	0.9980	1-250	0.10	0.90
	y = 353108 x + 2471750	0.9976	1-250		
Spirodiclofen	y = 45475.6 x + 176387	0.9990	1-250	0.15	1.03
	y = 43952.5 x + 194879	0.9984	1-250		
Sulfotep	y = 221374 x + 1231670	0.9973	1-250	0.12	0.99
	y = 223151 x + 1500600	0.9968	1-250		
Sulprofos	y = 76928.3 x + 768290	0.9974	1-250	0.63	0.90

	y = 85161.1 x + 763653	0.9976	1-250		
Tebuconazole	y = 140211 x + 641730	0.9985	1-250	0.07	0.90
	y = 156376 x + 1092590	0.9961	1-250		
Tebufenozide	y = 49185.9 x + 209616	0.9990	1-250	0.95	0.80
	y = 61535.3 x + 320772	0.9979	1-250		
Tebufenpyrad	y = 106143 x + 401472	0.9988	1-250	0.07	1.14
	y = 92916.7 x + 528019	0.9965	1-250		
Temephos	y = 340627 x + 2644300	0.9976	1-250	0.08	0.99
	y = 343536 x + 2610770	0.9969	1-250		
Terbufos	y = 12269.9 x + 148484	0.9947	2-250	2.67	0.96
	y = 12725.3 x + 140710	0.9965	2-250		
Tetrachlorvinphos	y = 28255.7 x + 229903	0.9970	1-250	0.06	0.80
	y = 35145.0 x + 256671	0.9972	1-250		
Tetraconazole	y = 145932 x + 795883	0.9974	1-250	0.33	0.80
	y = 182889 x + 950755	0.9989	1-250		
Thiacloprid	y = 165257 x + 26780.4	0.9998	1-100	0.43	0.25
	y = 649638 x + 824747	0.9996	1-100		
Thiamethoxam	y = 52103.0 x + 69765.9	0.9998	1-250	0.15	0.39
	y = 133709 x + 386705	0.9998	1-250		
Thiophanate-methyl	y = 248271 x + 1154090	0.9980	1-250	0.18	0.84
	y = 295481 x + 984774	0.9995	1-250		
Tolfenpyrad	y = 105060 x + 521093	0.9984	1-250	0.08	1.04
	y = 101463 x + 557008	0.9988	1-250		
Triadimefon	y = 49551.4 x + 537757	0.9971	2-250	1.63	1.05
	y = 46994.8 x + 450105	0.9987	2-250		
Triadimenol	y = 41818.0 x + 212857	0.9992	1-250	1.55	1.07
	y = 39055.2 x + 144867	0.9995	1-250		
Trifloxystrobin	y = 252629 x + 176424	0.9997	1-100	0.67	0.39

	y = 646293 x + 726873	0.9996	1-100		
Triticonazole	y = 96013.4 x + 92039.4	0.9999	1-250	0.30	0.97
	y = 99459.4 x + 431537	0.9986	1-250		
Uniconazole	y = 162925 x + 551645	0.9993	1-250	0.77	0.84
	y = 194158 x + 795576	0.9994	1-250		
Vitavax	y = 3070450 x + 78264.2	0.9990	1-100	0.08	0.88
	y = 3487430 x + 2053310	0.9985	1-100		

[1] Calibration curves are expressed as regression lines (y = a x + b), where y is the peak area and x is the concentration (μg L^{-1}), a is the slope, b is the intercept and R is the correlation coefficient.

[2] The first calibration curve for each pesticide represents the matrix-matched calibration curve.

[3] The second calibration curve for each pesticide represents the solvent standard calibration curve.

Table S4 The linearity, LOD and ME of each pesticide in FC.

Pesticides	[1]y=a x+b	R	Linear range, $\mu g\ L^{-1}$	LOD, $\mu g\ L^{-1}$	ME
Acephate	[2]y = 85843.1 x + 44764.7	0.9999	1-250	0.13	0.90
	[3]y = 95454.7 x + 126007	0.9998	1-250		
Acetamiprid	y = 76240.7 x - 10158.0	0.9992	1-250	0.14	0.35
	y = 216310 x + 859034	0.9996	1-250		
Alachlor	y = 1224780 x + 2898890	0.9990	1-100	1.07	0.93
	y = 1319150 x + 3664510	0.9956	1-100		
Atrazine	y = 142505 x + 721217	0.9970	1-250	0.44	0.87
	y = 163204 x + 1200030	0.9981	1-250		
Avermectin	y = 15608.7 x + 46863.3	0.9985	1-250	1.28	0.99
	y = 15804.3 x + 78893.4	0.9978	1-250		
Azaconazole	y = 203444 x + 851389	0.9994	1-250	0.80	0.77
	y = 264001 x + 1277940	0.9993	1-250		
Azamethiphos	y = 43326.2 x - 201892	0.9967	1-250	0.39	0.49
	y = 88694.2 x - 180974	0.9985	1-250		
Azoxystrobin	y = 627210 x + 836428	0.9996	1-100	0.82	0.83
	y = 758953 x + 1340200	0.9989	1-100		
Benalaxyl	y = 151326 x + 613130	0.9988	1-250	0.17	1.01
	y = 149996 x + 734635	0.9988	1-250		
Bendiocarb	y = 71733.1 x - 57901.8	0.9988	1-250	0.35	0.73
	y = 98646.2 x + 217493	0.9996	1-250		
Bitertanol	y = 12066.2 x + 31163.3	0.9992	1-250	1.58	1.10
	y = 10941.0 x + 27922.0	0.9991	1-250		
Bromuconazole	y = 29217.3 x + 182005	0.9978	1-250	2.20	0.82
	y = 35549.1 x + 205283	0.9991	1-250		
Buprofezin	y = 288072 x + 1755700	0.9976	1-250	0.05	0.97

	y = 296179 x + 1739250	0.9981	1-250		
Cadusafos	y = 282998 x + 560925	0.9970	1-250	0.61	0.92
	y = 305975 x + 1489500	0.9990	1-250		
Carbendazim	y = 222290 x + 377617	0.9998	1-250	0.19	0.69
	y = 320186 x + 1825120	0.9988	1-250		
Carbofuran	y = 163976 x - 282664	0.9990	1-250	0.28	0.52
	y = 315291 x + 1980730	0.9990	1-250		
Chlorantraniliprole	y = 54913.8 x + 203917	0.9994	1-250	0.49	0.90
	y = 60806.5 x + 374502	0.9984	1-250		
Chlorpyrifos	y = 1041230 x + 1438380	0.9989	1-100	0.11	0.89
	y = 1170180 x + 1250410	0.9991	1-100		
Chlortoluron	y = 152776 x + 513988	0.9996	1-250	0.41	0.68
	y = 224329 x + 1248420	0.9994	1-250		
Clothianidin	y = 29054.7 x - 6445.24	0.9994	1-250	0.21	0.44
	y = 66466.1 x + 259289	0.9995	1-250		
Coumaphos	y = 183781 x + 1287480	0.9983	1-250	0.12	0.98
	y = 187969 x + 1204260	0.9983	1-250		
Cyanofenphos	y = 34604.8 x + 227348	0.9982	1-250	0.28	0.91
	y = 38061.2 x + 194748	0.9990	1-250		
Cyazofamid	y = 53791.9 x + 237882	0.9991	1-250	0.13	0.64
	y = 84102.7 x + 400697	0.9995	1-250		
Cyproconazole	y = 147671 x + 515065	0.9994	1-250	0.09	0.91
	y = 161982 x + 841769	0.9989	1-250		
Dicrotophos	y = 56160.8 x + 18675.4	0.9998	1-250	0.20	0.98
	y = 57100.5 x + 80168.3	0.9999	1-250		
Difenoconazole	y = 150052 x + 427557	0.9995	1-250	0.15	1.02
	y = 147556 x + 675191	0.9984	1-250		
Dimethoate	y = 116512 x + 48130.9	0.9996	1-250	0.18	0.48

	y = 240641 x + 586878	0.9998	1-250		
Dimethomorph	y = 200599 x + 532745	0.9993	1-250	0.20	0.95
	y = 212219 x + 858828	0.9991	1-250		
Dimethyl phthalate	y = 57382.9 x + 742812	0.9963	1-250	0.71	0.46
	y = 125246 x + 2067560	0.9999	1-250		
Diniconazole	y = 50554.6 x + 40536.1	0.9998	1-250	0.29	0.93
	y = 54617.9 x + 134041	0.9997	1-250		
Dinotefuran	y = 60226.3 x + 137754	0.9998	1-250	0.18	0.95
	y = 63144.0 x + 193831	0.9997	1-250		
Emamectin Benzoate	y = 332491 x + 754358	0.9985	1-250	0.48	1.02
	y = 325660 x + 628673	0.9994	1-250		
Epoxiconazol	y = 83906.6 x + 857340	0.9981	1-250	0.18	0.73
	y = 114171 x + 775284	0.9975	1-250		
Ethiofencarb	y = 899696 x + 1917390	0.9974	1-100	0.64	0.61
	y = 1478020 x +2025200	0.9990	1-100		
Ethofenprox	y = 280218 x + 981199	0.9969	1-250	0.15	1.06
	y = 263965 x + 864543	0.9987	1-250		
Etoxazole	y = 752726 x + 1743730	0.9988	1-100	0.20	0.99
	y = 758833 x + 1867190	0.9985	1-100		
Fenamiphos	y = 332323 x + 3158540	0.9970	1-250	0.63	0.98
	y = 339535 x + 3259620	0.9975	1-250		
Fenamiphos sulfoxide	y = 85419.8 x + 22632.2	0.9993	1-250	0.49	0.64
	y = 133042 x + 550303	0.9994	1-250		
Fenbuconazole	y = 59843.5 x + 248121	0.9993	1-250	0.19	0.89
	y = 67466.4 x + 287280	0.9996	1-250		
Fenpropathrin	y = 8840.35 x + 53147.8	0.9987	2-250	1.43	0.88
	y = 10089.5 x + 53148.5	0.9988	2-250		
Fipronil	y = 10506.4 x + 10164.4	0.9996	1-250	0.78	0.97

	y = 10861.0 x + 47921.3	0.9971	1-250		
Fipronil-sulfone	y = 159820 x + 124915	0.9999	1-250	0.25	0.85
	y = 187287 x + 361756	0.9993	1-250		
Fludioxonil	y = 29234.3 x + 226935	0.9973	1-250	0.06	0.93
	y = 31485.4 x + 274109	0.9972	1-250		
Fluopyram	y = 159531 x + 995164	0.9979	1-250	0.30	0.84
	y = 189716 x + 1098190	0.9988	1-250		
Fluoxastrobin	y = 275546 x + 1622720	0.9988	1-250	0.46	0.90
	y = 305857 x + 2315090	0.9976	1-250		
Flusilazole	y = 133442 x + 550469	0.9992	1-250	0.14	0.91
	y = 146458 x + 721624	0.9989	1-250		
Flutolanil	y = 301441 x + 2412670	0.9966	1-250	0.30	0.90
	y = 333385 x + 2389380	0.9984	1-250		
Flutriafol	y = 29695.0 x + 44353.6	0.9997	1-250	0.31	0.71
	y = 41757.2 x + 226352	0.9991	1-250		
Hexaconazole	y = 65846.9 x + 140982	0.9995	1-250	0.25	1.02
	y = 64745.7 x + 399719	0.9983	1-250		
Hexaflumuron	y = 340100 x + 2463060	0.9967	1-250	0.17	1.01
	y = 337835 x + 2789150	0.9972	1-250		
Hexazinone	y = 257706 x + 232980	0.9979	1-100	0.27	0.34
	y = 768340 x + 1387410	0.9996	1-100		
3-Hydroxy-carbofuran	y = 8340.17 x - 18527.5	0.9983	2-250	1.76	0.39
	y = 21261.4 x + 67880.3	0.9998	2-250		
Imazalil	y = 75837.4 x + 448874	0.9980	1-250	0.18	0.82
	y = 92868.5 x + 343302	0.9988	1-250		
Imidacloprid	y = 45032.5 x + 101951	0.9998	1-250	0.23	0.57
	y = 79673.5 x + 342956	0.9992	1-250		
Indoxacarb	y = 19691.6 x + 77596.2	0.9994	1-250	0.55	1.06

	y = 18648.3 x + 123693	0.9969	1-250		
Ipconazole	y = 156239 x + 364285	0.9994	1-250	0.15	0.88
	y = 178395 x + 768412	0.9985	1-250		
Isoprocarb	y = 117130 x + 595832	0.9978	1-250	0.43	0.92
	y = 126739 x + 933704	0.9986	1-250		
Kresoxim-methyl	y = 43902.0 x + 226791	0.9974	1-250	0.33	1.01
	y = 43423.6 x + 289101	0.9974	1-250		
Lufenuron	y = 10727.3 x + 121844	0.9973	1-250	0.84	0.92
	y = 11683.4 x + 152160	0.9943	1-250		
Machette	y = 197885 x + 1185040	0.9981	1-250	0.14	1.01
	y = 196230 x + 1188680	0.9978	1-250		
Malaoxon	y = 512867 x - 237645	0.9969	1-100	0.59	0.66
	y = 777428 x + 1903040	0.9992	1-100		
Malathion	y = 133554 x + 1216780	0.9977	1-250	0.82	0.98
	y = 135705 x + 1538610	0.9979	1-250		
Mepronil	y = 2978480 x + 2368610	0.9977	1-20/	0.15	0.78
	y = 3839890 x + 2734280	0.9993	1-100		
Metalaxyl	y = 627339 x + 747054	0.9979	1-100	0.25	0.79
	y = 797536 x + 1731510	0.9991	1-100		
Metalaxyl-M	y = 640190 x + 834499	0.9983	1-100	0.74	0.81
	y = 792866 x + 1973550	0.9990	1-100		
Methidathion	y = 27447.9 x + 109632	0.9968	1-250	0.60	0.83
	y = 33184.7 x + 229958	0.9964	1-250		
Methomyl	y = 67742.8 x + 56830.1	0.9999	1-250	0.13	0.95
	y = 71661.7 x + 193993	0.9996	1-250		
Methoprene	y = 27379.8 x + 67417.1	0.9997	1-250	0.57	1.01
	y = 27018.2 x + 138200	0.9980	1-250		
Methoxyfenozide	y = 65893.0 x + 358492	0.9989	1-250	0.26	0.90

	y = 72975.4 x + 455840	0.9984	1-250		
Mevinphos	y = 29522.0 x - 106846	0.9981	2-250	0.80	0.42
	y = 70676.6 x + 155188	0.9997	2-250		
Monocrotophos	y = 37547.1 x - 9640.81	0.9996	1-250	0.39	1.03
	y = 36326.6 x + 59398.2	0.9997	1-250		
Myclobutanil	y = 59482.8 x + 402701	0.9970	1-250	0.23	0.97
	y = 61274.2 x + 590971	0.9962	1-250		
Omethoate	y = 87078.9 x + 143419	0.9997	1-250	0.09	1.00
	y = 86878.8 x + 240660	0.9995	1-250		
Paclobutrazol	y = 299194 x + 1536100	0.9986	1-250	0.18	0.82
	y = 366191 x + 2096590	0.9988	1-250		
Penconazole	y = 90931.8 x + 493087	0.9980	1-250	0.11	0.98
	y = 92647.8 x + 648178	0.9979	1-250		
Phenthoate	y = 123757 x + 865286	0.9984	1-250	0.05	0.82
	y = 150919 x + 1088000	0.9984	1-250		
Phorate	y = 31248.4 x + 51345.5	0.9998	1-250	0.11	0.82
	y = 37916.5 x + 75618.0	0.9993	1-250		
Phorate sulfone	y = 30371.0 x + 142057	0.9995	1-250	0.83	0.76
	y = 39917.1 x + 287688	0.9986	1-250		
Phosalone	y = 1245400 x + 2215450	0.9986	1-100	1.56	0.94
	y = 1325190 x + 2320790	0.9984	1-100		
Phosfolan	y = 242047 x - 414556	0.9988	1-250	0.15	0.68
	y = 353707 x + 1927950	0.9993	1-250		
Phosphamidon	y = 96171.0 x - 143984	0.9989	1-250	0.22	0.67
	y = 143170 x + 102740	0.9998	1-250		
Piperonyl butoxide	y = 313403 x + 1740420	0.9987	1-250	0.24	1.00
	y = 313792 x + 1849940	0.9976	1-250		
Pirimicarb	y = 310620 x + 606324	0.9998	1-250	0.11	0.91

	y = 339671 x + 1637580	0.9994	1-250		
Pirimiphos-methyl	y = 280682 x + 574881	0.9996	1-250	0.09	0.87
	y = 321052 x + 1858120	0.9977	1-250		
Prochloraz	y = 145779 x + 590929	0.9988	1-250	0.18	0.95
	y = 153835 x + 1028100	0.9984	1-250		
Profenofos	y = 96052.8 x + 575749	0.9987	1-250	0.09	0.95
	y = 100949 x + 465548	0.9989	1-250		
Propamocarb	y = 258519 x - 148645	0.9997	1-250	0.16	1.25
	y = 206167 x - 219273	0.9996	1-250		
Propiconazole	y = 91728.3 x + 604260	0.9980	1-250	0.17	0.93
	y = 98529.3 x + 664585	0.9977	1-250		
Pyraclostrobin	y = 170554 x + 628197	0.9989	1-250	0.21	0.94
	y = 181948 x + 753832	0.9993	1-250		
Pyridaben	y = 347266 x + 2093940	0.9977	1-250	0.15	0.96
	y = 360922 x + 3184610	0.9919	1-250		
Pyridafenthion	y = 157547 x + 1223090	0.9970	1-250	0.86	0.93
	y = 170224 x + 1467230	0.9976	1-250		
Pyrimitate	y = 940503 x + 1626720	0.9977	1-100	0.13	0.75
	y = 1245920 x + 367730	0.9997	1-100		
Pyrisoxazole	y = 24312.0 x + 74387.8	0.9982	1-250	0.22	0.93
	y = 26076.3 x + 108653	0.9987	1-250		
Simeconazole	y = 299211 x + 1492180	0.9989	1-250	0.11	0.85
	y = 353108 x + 2471750	0.9976	1-250		
Spirodiclofen	y = 43942.2 x + 111706	0.9985	1-250	0.12	1.00
	y = 43952.5 x + 194879	0.9984	1-250		
Sulfotep	y = 201714 x + 1149350	0.9986	1-250	0.06	0.90
	y = 223151 x + 1500600	0.9968	1-250		
Sulprofos	y = 81372.2 x + 797934	0.9968	1-250	0.31	0.96

	y = 85161.1 x + 763653	0.9976	1-250		
Tebuconazole	y = 147481 x + 469067	0.9973	1-250	0.17	0.94
	y = 156376 x + 1092590	0.9961	1-250		
Tebufenozide	y = 56077.0 x + 118687	0.9994	1-250	1.68	0.91
	y = 61535.3 x + 320772	0.9979	1-250		
Tebufenpyrad	y = 94525.5 x + 378735	0.9993	1-250	0.16	1.02
	y = 92916.7 x + 528019	0.9965	1-250		
Temephos	y = 326104 x + 2560530	0.9981	1-250	0.11	0.95
	y = 343536 x + 2610770	0.9969	1-250		
Terbufos	y = 12673.1 x + 140017	0.9960	2-250	1.01	1.00
	y = 12725.3 x + 140710	0.9965	2-250		
Tetrachlorvinphos	y = 34401.6 x + 178660	0.9987	1-250	0.12	0.98
	y = 35145.0 x + 256671	0.9972	1-250		
Tetraconazole	y = 164884 x + 297149	0.9997	1-250	0.07	0.90
	y = 182889 x + 950755	0.9989	1-250		
Thiacloprid	y = 335009 x + 366369	0.9994	1-100	0.33	0.52
	y = 649638 x + 824747	0.9996	1-100		
Thiamethoxam	y = 114790 x + 259243	0.9999	1-250	0.08	0.86
	y = 133709 x + 386705	0.9998	1-250		
Thiophanate-methyl	y = 143721 x - 266221	0.9987	1-250	0.12	0.49
	y = 295481 x + 984774	0.9995	1-250		
Tolfenpyrad	y = 90058.0 x + 612117	0.9981	1-250	0.11	0.89
	y = 101463 x + 557008	0.9988	1-250		
Triadimefon	y = 43441.4 x + 427493	0.9984	2-250	1.52	0.92
	y = 46994.8 x + 450105	0.9987	2-250		
Triadimenol	y = 37087.1 x + 135671	0.9990	1-250	1.32	0.95
	y = 39055.2 x + 144867	0.9995	1-250		
Trifloxystrobin	y = 462954 x + 610327	0.9996	1-100	1.22	0.72

	y = 646293 x + 726873	0.9996	1-100		
Triticonazole	y = 76368.4 x + 298187	0.9993	1-250	0.23	0.77
	y = 99459.4 x + 431537	0.9986	1-250		
Uniconazole	y = 165721 x + 467852	0.9995	1-250	0.12	0.85
	y = 194158 x + 795576	0.9994	1-250		
Vitavax	y = 1406400 x + 1784080	0.9966	1-100	0.12	0.40
	y = 3487430 x + 2053310	0.9985	1-100		

[1] Calibration curves are expressed as regression lines (y = a x + b), where y is the peak area and x is the concentration (μg L^{-1}), a is the slope, b is the intercept and R is the correlation coefficient.

[2] The first calibration curve for each pesticide represents the matrix-matched calibration curve.

[3] The second calibration curve for each pesticide represents the solvent standard calibration curve.

Table S5 The linearity, LOD and ME of each pesticide in FD.

Pesticides	[1]y=a x+b	R	Linear range, μg L^{-1}	LOD, μg L^{-1}	ME
Acephate	[2]y = 67806.2 x - 7139.02	0.9996	1-250	0.16	0.71
	[3]y = 95454.7 x + 126007	0.9998	1-250		
Acetamiprid	y = 93931.6 x + 220641	0.9997	1-250	0.18	0.43
	y = 216310 x + 859034	0.9996	1-250		
Alachlor	y = 1253120 x + 2690540	0.9987	1-100	0.88	0.95
	y = 1319150 x + 3664510	0.9956	1-100		
Atrazine	y = 118439 x + 640960	0.9972	1-250	0.29	0.73
	y = 163204 x + 1200030	0.9981	1-250		
Avermectin	y = 11652.9 x + 70367.9	0.9977	1-250	1.26	0.74
	y = 15804.3 x + 78893.4	0.9978	1-250		
Azaconazole	y = 227372 x + 1156260	0.9972	1-250	0.68	0.86
	y = 264001 x + 1277940	0.9993	1-250		
Azamethiphos	y = 51824.4 x + 173020	0.9971	1-250	0.26	0.58
	y = 88694.2 x - 180974	0.9985	1-250		
Azoxystrobin	y = 663961 x + 712863	0.9997	1-100	0.83	0.87
	y = 758953 x + 1340200	0.9989	1-100		
Benalaxyl	y = 117499 x + 617712	0.9986	1-250	0.22	0.78
	y = 149996 x + 734635	0.9988	1-250		
Bendiocarb	y = 53028.4 x + 199417	0.9993	1-250	0.30	0.54
	y = 98646.2 x + 217493	0.9996	1-250		
Bitertanol	y = 11914.9 x + 38177.2	0.9992	1-250	1.07	1.09
	y = 10941.0 x + 27922.0	0.9991	1-250		
Bromuconazole	y = 36174.5 x + 54471.2	0.9992	1-250	2.59	1.02
	y = 35549.1 x + 205283	0.9991	1-250		
Buprofezin	y = 294688 x + 1536460	0.9962	1-250	0.05	0.99

	y = 296179 x + 1739250	0.9981	1-250		
Cadusafos	y = 269756 x + 1103120	0.9992	1-250	1.01	0.88
	y = 305975 x + 1489500	0.9990	1-250		
Carbendazim	y = 225306 x + 648281	0.9996	1-250	0.21	0.70
	y = 320186 x + 1825120	0.9988	1-250		
Carbofuran	y = 129359 x + 474898	0.9976	1-250	0.32	0.41
	y = 315291 x + 1980730	0.9990	1-250		
Chlorantraniliprole	y = 55423.8 x + 237239	0.9990	1-250	0.83	0.91
	y = 60806.5 x + 374502	0.9984	1-250		
Chlorpyrifos	y = 984924 x + 1727480	0.9980	1-100	0.15	0.84
	y = 1170180 x + 1250410	0.9991	1-100		
Chlortoluron	y = 159242 x + 772193	0.9985	1-250	0.22	0.71
	y = 224329 x + 1248420	0.9994	1-250		
Clothianidin	y = 41334.4 x + 33322.1	0.9998	1-250	0.11	0.62
	y = 66466.1 x + 259289	0.9995	1-250		
Coumaphos	y = 160608 x + 1044170	0.9985	1-250	0.07	0.85
	y = 187969 x + 1204260	0.9983	1-250		
Cyanofenphos	y = 33187.8 x + 234269	0.9978	1-250	0.18	0.87
	y = 38061.2 x + 194748	0.9990	1-250		
Cyazofamid	y = 50740.0 x + 388912	0.9976	1-250	0.11	0.60
	y = 84102.7 x + 400697	0.9995	1-250		
Cyproconazole	y = 142246 x + 671284	0.9977	1-250	0.14	0.88
	y = 161982 x + 841769	0.9989	1-250		
Dicrotophos	y = 54428.7 x + 30376.8	0.9997	1-250	0.19	0.95
	y = 57100.5 x + 80168.3	0.9999	1-250		
Difenoconazole	y = 148109 x + 180322	0.9999	1-250	0.10	1.00
	y = 147556 x + 675191	0.9984	1-250		
Dimethoate	y = 126041 x + 440503	0.9988	1-250	0.29	0.52

	y = 240641 x + 586878	0.9998	1-250		
Dimethomorph	y = 208965 x + 580490	0.9990	1-250	0.14	0.98
	y = 212219 x + 858828	0.9991	1-250		
Dimethyl phthalate	y = 63283.3 x + 1927150	0.9974	1-250	1.33	0.51
	y = 125246 x + 2067560	0.9999	1-250		
Diniconazole	y = 48457.6 x + 198578	0.9974	1-250	0.19	0.89
	y = 54617.9 x + 134041	0.9997	1-250		
Dinotefuran	y = 58085.6 x + 49799.7	0.9998	1-250	0.13	0.92
	y = 63144.0 x + 193831	0.9997	1-250		
Emamectin Benzoate	y = 333395 x + 688468	0.9989	1-250	0.44	1.02
	y = 325660 x + 628673	0.9994	1-250		
Epoxiconazol	y = 116415 x + 614516	0.9991	1-250	0.18	1.02
	y = 114171 x + 775284	0.9975	1-250		
Ethiofencarb	y = 1105010 x + 1035670	0.9983	1-100	0.44	0.75
	y = 1478020 x + 2025200	0.9990	1-100		
Ethofenprox	y = 267762 x + 740671	0.9993	1-250	0.12	1.01
	y = 263965 x + 864543	0.9987	1-250		
Etoxazole	y = 737336 x + 1671050	0.9989	1-100	0.24	0.97
	y = 758833 x + 1867190	0.9985	1-100		
Fenamiphos	y = 325909 x + 3253420	0.9962	1-250	0.58	0.96
	y = 339535 x + 3259620	0.9975	1-250		
Fenamiphos sulfoxide	y = 79340.1 x + 396890	0.9976	1-250	0.28	0.60
	y = 133042 x + 550303	0.9994	1-250		
Fenbuconazole	y = 72215.0 x + 42991.2	0.9991	1-250	0.25	1.07
	y = 67466.4 x + 287280	0.9996	1-250		
Fenpropathrin	y = 9545.82 x + 64136.3	0.9974	2-250	0.90	0.95
	y = 10089.5 x + 53148.5	0.9988	2-250		
Fipronil	y = 10696.3 x + 16162.5	0.9994	1-250	0.56	0.98

	y = 10861.0 x + 47921.3	0.9971	1-250		
Fipronil-sulfone	y = 182705 x - 306552	0.9980	1-250	0.53	0.98
	y = 187287 x + 361756	0.9993	1-250		
Fludioxonil	y = 30562.0 x + 222403	0.9972	1-250	0.10	0.97
	y = 31485.4 x + 274109	0.9972	1-250		
Fluopyram	y = 174970 x + 650091	0.9989	1-250	0.16	0.92
	y = 189716 x + 1098190	0.9988	1-250		
Fluoxastrobin	y = 289759 x + 1491470	0.9987	1-250	0.20	0.95
	y = 305857 x + 2315090	0.9976	1-250		
Flusilazole	y = 128148 x + 444292	0.9994	1-250	0.21	0.87
	y = 146458 x + 721624	0.9989	1-250		
Flutolanil	y = 313102 x + 1185610	0.9981	1-250	0.50	0.94
	y = 333385 x + 2389380	0.9984	1-250		
Flutriafol	y = 27789.9 x + 142913	0.9989	1-250	0.32	0.67
	y = 41757.2 x + 226352	0.9991	1-250		
Hexaconazole	y = 53825.9 x + 299357	0.9982	1-250	0.24	0.83
	y = 64745.7 x + 399719	0.9983	1-250		
Hexaflumuron	y = 318013 x + 2253280	0.9965	1-250	0.11	0.94
	y = 337835 x + 2789150	0.9972	1-250		
Hexazinone	y = 244642 x + 80326.2	0.9985	1-100	0.42	0.32
	y = 768340 x + 1387410	0.9996	1-100		
3-Hydroxy-carbofuran	y = 10814.3 x + 19959.1	0.9999	2-250	0.89	0.51
	y = 21261.4 x + 67880.3	0.9998	2-250		
Imazalil	y = 84788.7 x - 67927.8	0.9996	1-250	0.90	0.91
	y = 92868.5 x + 343302	0.9988	1-250		
Imidacloprid	y = 57399.2 x + 197573	0.9994	1-250	0.14	0.72
	y = 79673.5 x + 342956	0.9992	1-250		
Indoxacarb	y = 21757.7 x + 41414.0	0.9995	1-250	0.29	1.17

	y = 18648.3 x + 123693	0.9969	1-250		
Ipconazole	y = 172626 x + 73562.0	0.9997	1-250	0.19	0.97
	y = 178395 x + 768412	0.9985	1-250		
Isoprocarb	y = 99757.8 x + 741441	0.9961	1-250	0.28	0.79
	y = 126739 x + 933704	0.9986	1-250		
Kresoxim-methyl	y = 46639.2 x + 93053.1	0.9991	1-250	0.61	1.07
	y = 43423.6 x + 289101	0.9974	1-250		
Lufenuron	y = 10389.1 x + 127797	0.9963	1-250	1.52	0.89
	y = 11683.4 x + 152160	0.9943	1-250		
Machette	y = 194649 x + 871311	0.9993	1-250	0.24	0.99
	y = 196230 x + 1188680	0.9978	1-250		
Malaoxon	y = 443351 x + 221032	0.9980	1-100	0.24	0.57
	y = 777428 x + 1903040	0.9992	1-100		
Malathion	y = 112295 x + 1533350	0.9961	1-250	0.98	0.83
	y = 135705 x + 1538610	0.9979	1-250		
Mepronil	y = 3451270 x + 380017	0.9966	1-100	0.24	0.90
	y = 3839890 x + 2734280	0.9993	1-100		
Metalaxyl	y = 639745 x + 1188510	0.9983	1-100	0.52	0.80
	y = 797536 x + 1731510	0.9991	1-100		
Metalaxyl-M	y = 648931 x + 1147860	0.9987	1-100	0.31	0.82
	y = 792866 x + 1973550	0.9990	1-100		
Methidathion	y = 24284.8 x + 181172	0.9960	1-250	1.71	0.73
	y = 33184.7 x + 229958	0.9964	1-250		
Methomyl	y = 63359.5 x + 60103.7	0.9997	1-250	0.07	0.88
	y = 71661.7 x + 193993	0.9996	1-250		
Methoprene	y = 25603.7 x + 94951.4	0.9988	1-250	0.53	0.95
	y = 27018.2 x + 138200	0.9980	1-250		
Methoxyfenozide	y = 77538.8 x + 301497	0.9987	2-250	0.55	1.07

	y = 72520.7 x + 535238	0.9985	2-250		
Mevinphos	y = 36183.8 x + 85649.8	0.9992	2-250	0.44	0.51
	y = 70676.6 x + 155188	0.9997	2-250		
Monocrotophos	y = 33127.1 x + 53348.9	0.9997	1-250	0.37	0.91
	y = 36326.6 x + 59398.2	0.9997	1-250		
Myclobutanil	y = 61544.1 x + 488798	0.9963	1-250	0.32	1.00
	y = 61274.2 x + 590971	0.9962	1-250		
Omethoate	y = 81508.3 x + 61656.9	0.9998	1-250	0.10	0.94
	y = 86878.8 x + 240660	0.9995	1-250		
Paclobutrazol	y = 315465 x + 1293340	0.9980	1-250	0.10	0.86
	y = 366191 x + 2096590	0.9988	1-250		
Penconazole	y = 72818.3 x + 354940	0.9994	1-250	0.23	0.79
	y = 92647.8 x + 648178	0.9979	1-250		
Phenthoate	y = 117291 x + 869493	0.9979	1-250	0.15	0.78
	y = 150919 x + 1088000	0.9984	1-250		
Phorate	y = 25567.0 x + 68696.6	0.9994	1-250	0.24	0.67
	y = 37916.5 x + 75618.0	0.9993	1-250		
Phorate sulfone	y = 27847.0 x + 345853	0.9978	5-250	0.93	0.71
	y = 39237.9 x + 407020	0.9994	5-250		
Phosalone	y = 1156490 x + 1415210	0.9992	1-100	0.67	0.87
	y = 1325190 x + 2320790	0.9984	1-100		
Phosfolan	y = 277856 x + 697510	0.9990	1-250	0.18	0.79
	y = 353707 x + 1927950	0.9993	1-250		
Phosphamidon	y = 99382.7 x + 129213	0.9996	1-250	0.28	0.69
	y = 143170 x + 102740	0.9998	1-250		
Piperonyl butoxide	y = 314310 x + 1620890	0.9982	1-250	0.12	1.00
	y = 313792 x + 1849940	0.9976	1-250		
Pirimicarb	y = 303829 x + 716498	0.9986	1-250	0.08	0.89

	y = 339671 x + 1637580	0.9994	1-250		
Pirimiphos-methyl	y = 261259 x + 559621	0.9988	1-250	0.16	0.81
	y = 321052 x + 1858120	0.9977	1-250		
Prochloraz	y = 154104 x + 130468	0.9999	1-250	0.18	1.00
	y = 153835 x + 1028100	0.9984	1-250		
Profenofos	y = 92962.4 x + 595027	0.9982	1-250	0.13	0.92
	y = 100949 x + 465548	0.9989	1-250		
Propamocarb	y = 248360 x - 196874	0.9995	1-250	0.14	1.20
	y = 206167 x - 219273	0.9996	1-250		
Propiconazole	y = 96650.3 x + 581100	0.9971	1-250	0.15	0.98
	y = 98529.3 x + 664585	0.9977	1-250		
Pyraclostrobin	y = 194122 x + 216011	0.9998	1-250	0.19	1.07
	y = 181948 x + 753832	0.9993	1-250		
Pyridaben	y = 343777 x + 1888040	0.9988	1-250	0.13	0.95
	y = 360922 x + 3184610	0.9919	1-250		
Pyridafenthion	y = 189532 x + 1166240	0.9963	1-250	1.10	1.11
	y = 170224 x + 1467230	0.9976	1-250		
Pyrimitate	y = 1011580 x + 599577	0.9995	1-100	0.31	0.81
	y = 1245920 x + 367730	0.9997	1-100		
Pyrisoxazole	y = 24746.1 x + 59031.7	0.9995	1-250	0.24	0.95
	y = 26076.3 x + 108653	0.9987	1-250		
Simeconazole	y = 317073 x + 1264780	0.9986	1-250	0.15	0.90
	y = 353108 x + 2471750	0.9976	1-250		
Spirodiclofen	y = 43548.4 x + 109356	0.9993	1-250	0.21	0.99
	y = 43952.5 x + 194879	0.9984	1-250		
Sulfotep	y = 236375 x + 686357	0.9982	1-250	0.12	1.06
	y = 223151 x + 1500600	0.9968	1-250		
Sulprofos	y = 81204.1 x + 796898	0.9970	1-250	0.42	0.95

	y = 85161.1 x + 763653	0.9976	1-250		
Tebuconazole	y = 127516 x + 486203	0.9990	1-250	0.13	0.82
	y = 156376 x + 1092590	0.9961	1-250		
Tebufenozide	y = 51176.4 x + 279101	0.9978	1-250	0.44	0.83
	y = 61535.3 x + 320772	0.9979	1-250		
Tebufenpyrad	y = 93311.2 x + 390575	0.9985	1-250	0.18	1.00
	y = 92916.7 x + 528019	0.9965	1-250		
Temephos	y = 337370 x + 2728750	0.9965	1-250	0.10	0.98
	y = 343536 x + 2610770	0.9969	1-250		
Terbufos	y = 12232.0 x + 133083	0.9965	2-250	1.62	0.96
	y = 12725.3 x + 140710	0.9965	2-250		
Tetrachlorvinphos	y = 29380.5 x + 243573	0.9968	1-250	0.03	0.84
	y = 35145.0 x + 256671	0.9972	1-250		
Tetraconazole	y = 150468 x + 365481	0.9993	1-250	0.25	0.82
	y = 182889 x + 950755	0.9989	1-250		
Thiacloprid	y = 418696 x + 452817	0.9993	1-100	0.88	0.64
	y = 649638 x + 824747	0.9996	1-100		
Thiamethoxam	y = 114170 x + 118094	0.9998	1-250	0.04	0.85
	y = 133709 x + 386705	0.9998	1-250		
Thiophanate-methyl	y = 145929 x + 725037	0.9979	1-250	0.11	0.49
	y = 295481 x + 984774	0.9995	1-250		
Tolfenpyrad	y = 93800.8 x + 576177	0.9978	1-250	0.06	0.92
	y = 101463 x + 557008	0.9988	1-250		
Triadimefon	y = 46025.2 x + 495280	0.9983	2-250	1.77	0.98
	y = 46994.8 x + 450105	0.9987	2-250		
Triadimenol	y = 37972.1 x + 125791	0.9992	1-250	0.96	0.97
	y = 39055.2 x + 144867	0.9995	1-250		
Trifloxystrobin	y = 376473 x + 619709	0.9993	1-100	0.62	0.58

	y = 646293 x + 726873	0.9996	1-100		
Triticonazole	y = 100310 x - 66743.8	0.9993	1-250	0.77	1.01
	y = 99459.4 x + 431537	0.9986	1-250		
Uniconazole	y = 156093 x + 718396	0.9973	1-250	0.17	0.80
	y = 194158 x + 795576	0.9994	1-250		
Vitavax	y = 1694970 x - 575122	0.9995	1-100	0.21	0.49
	y = 3487430 x + 2053310	0.9985	1-100		

[1] Calibration curves are expressed as regression lines (y = a x + b), where y is the peak area and x is the concentration (μg L^{-1}), a is the slope, b is the intercept and R is the correlation coefficient.

[2] The first calibration curve for each pesticide represents the matrix-matched calibration curve.

[3] The second calibration curve for each pesticide represents the solvent standard calibration curve.

Table S6 Recoveries of the pesticides at spiked concentrations of 30, 120, and 240 μg kg^{-1} in the FT samples.

Spiked concentration	30 μg kg^{-1}		120 μg kg^{-1}		240 μg kg^{-1}	
Pesticides	Recovery(%)	RSD(%)	Recovery(%)	RSD(%)	Recovery(%)	RSD(%)
Acephate	76.2	5	83.7	0.4	75.7	2
Acetamiprid	82.4	3	81.9	6	75.5	3
Alachlor	97.6	3	99.2	2	93.7	0.5
Atrazine	99.5	5	93.9	2	87.0	2
Avermectin	90.8	5	103	3	77.5	2
Azaconazole	93.6	7	92.0	3	85.5	3
Azamethiphos	85.8	2	96.4	8	76.4	5
Azoxystrobin	98.8	5	94.4	4	86.7	4
Benalaxyl	91.5	2	102	0.4	97.6	4
Bendiocarb	75.3	5	105	9	93.8	1
Bitertanol	103	7	98.6	2	93.6	3
Bromuconazole	93.4	8	92.4	3	86.5	6
Buprofezin	102	2	96.2	1	95.5	7
Cadusafos	93.0	5	99.5	3	102	4
Carbendazim	109	20	81.3	8	79.6	7
Carbofuran	71.1	13	95.3	0.4	88.3	3
Chlorantraniliprole	84.1	18	81.3	3	91.9	8
Chlorpyrifos	96.7	1	94.8	1	88.5	3
Chlortoluron	87.1	6	73.9	5	78.0	2
Clothianidin	95.0	9	83.2	3	85.2	3
Coumaphos	73.7	5	70.3	4	73.1	5
Cyanofenphos	91.0	2	105	4	94.0	4
Cyazofamid	95.1	1	93.3	5	93.5	3
Cyproconazole	103	2	95.4	0.6	94.9	2

Dicrotophos	95.6	5	96.7	5	93.0	4
Difenoconazole	92.7	5	97.1	2	90.5	0.5
Dimethoate	80.7	9	81.0	10	80.3	5
Dimethomorph	95.9	3	100	0.6	90.2	3
Dimethyl phthalate	90.6	3	98.2	9	78.4	3
Diniconazole	97.9	5	85.5	2	93.9	3
Dinotefuran	83.6	2	93.7	2	87.0	2
Emamectin Benzoate	88.6	1	82.9	4	81.8	6
Epoxiconazol	108	7	95.5	1	93.8	4
Ethiofencarb	71.0	2	94.5	9	90.9	11
Ethofenprox	95.1	3	80.9	1	71.8	2
Etoxazole	99.6	2	96.5	3	90.2	2
Fenamiphos	96.4	6	101	2	101	3
Fenamiphos sulfoxide	77.0	3	93.7	6	92.0	5
Fenbuconazole	108	12	108	2	90.2	5
Fenpropathrin	101	3	92.0	9	97.6	12
Fipronil	111	18	103	9	95.6	2
Fipronil-sulfone	93.6	8	104	2	99.5	2
Fludioxonil	84.4	7	82.8	4	78.5	6
Fluopyram	99.8	6	107	3	100	1
Fluoxastrobin	109	4	103	0.8	102	3
Flusilazole	93.1	8	101	2	93.4	2
Flutolanil	93.7	3	99.8	4	93.7	8
Flutriafol	96.7	6	105	4	97.5	3
Hexaconazole	96.7	8	99.3	1	99.1	5
Hexaflumuron	96.9	4	103	3	101	0.9
Hexazinone	87.3	6	97.2	10	86.7	2
3-Hydroxy-carbofuran	91.0	18	88.9	10	70.7	3

Imazalil	92.4	4	94.6	1	82.9	4
Imidacloprid	86.1	16	89.7	3	86.5	4
Indoxacarb	100	5	107	5	92.8	8
Ipconazole	97.5	4	90.7	2	83.6	7
Isoprocarb	97.8	7	95.0	4	91.0	2
Kresoxim-methyl	112	6	101	8	91.0	11
Lufenuron	87.1	9	80.6	11	77.8	3
Machette	99.7	7	96.3	3	95.8	0.2
Malaoxon	88.3	4	97.4	9	91.2	3
Malathion	91.2	8	98.8	6	96.3	4
Mepronil	87.2	2	94.0	3	87.4	3
Metalaxyl	97.3	3	90.7	4	90.6	5
Metalaxyl-M	94.1	1	93.8	4	91.3	4
Methidathion	104	6	92.1	0.9	98.0	6
Methomyl	95.6	4	91.0	0.7	90.1	1
Methoprene	88.4	4	98.8	6	86.0	0.2
Methoxyfenozide	94.0	2	96.9	8	94.9	4
Mevinphos	68.5	5	93.0	7	78.5	6
Monocrotophos	105	9	90.8	2	90.2	0.7
Myclobutanil	109	3	93.7	3	94.6	4
Omethoate	85.7	3	86.3	2	84.3	4
Paclobutrazol	93.5	2	97.8	2	96.7	2
Penconazole	101	0.7	98.2	2	98.0	6
Phenthoate	88.8	6	101	1	96.2	5
Phorate	100	5	101	3	108	12
Phorate sulfone	99.7	4	99.4	8	95.7	1
Phosalone	90.0	6	88.9	1	86.6	3
Phosfolan	109	4	88.8	4	88.9	2

Phosphamidon	93.7	11	90.4	1	89.5	8
Piperonyl butoxide	99.4	1	97.9	2	95.6	2
Pirimicarb	95.7	8	96.7	3	98.3	1
Pirimiphos-methyl	94.0	4	98.2	3	92.5	3
Prochloraz	66.2	5	74.5	4	69.8	4
Profenofos	94.9	7	96.9	6	96.1	6
Propamocarb	76.7	2	75.3	6	72.1	0.1
Propiconazole	97.6	2	99.7	5	94.1	1
Pyraclostrobin	78.6	17	70.0	4	61.2	5
Pyridaben	97.8	2	96.5	1	84.9	0.7
Pyridafenthion	119	10	85.3	0.7	91.4	4
Pyrimitate	97.6	4	95.7	3	89.8	6
Pyrisoxazole	110	4	92.6	5	94.4	8
Simeconazole	95.6	5	106	2	89.0	1
Spirodiclofen	84.8	3	91.7	3	94.0	2
Sulfotep	95.3	5	104	5	97.0	7
Sulprofos	91.1	4	94.2	2	89.2	5
Tebuconazole	85.2	5	92.0	4	96.5	4
Tebufenozide	91.8	11	102	6	91.7	0.8
Tebufenpyrad	107	3	97.9	3	93.6	3
Temephos	96.6	5	93.6	4	91.7	2
Terbufos	97.2	2	109	7	97.1	1
Tetrachlorvinphos	95.4	10	97.9	3	92.5	1
Tetraconazole	96.5	2	101	4	92.8	4
Thiacloprid	83.4	7	93.0	8	77.7	6
Thiamethoxam	93.7	6	92.6	4	83.7	5
Thiophanate-methyl	93.9	7	82.5	2	79.6	6
Tolfenpyrad	88.8	7	83.1	6	76.8	5

Triadimefon	101	7	106	2	101	5
Triadimenol	112	0.9	95.8	5	96.0	3
Trifloxystrobin	109	7	98.8	1	93.3	7
Triticonazole	108	17	98.1	1	90.7	5
Uniconazole	101	2	94.7	0.4	93.6	4
Vitavax	90.2	6	84.5	5	70.6	3

Table S7 Recoveries of the pesticides at spiked concentrations of 30, 120, and 240 μg kg^{-1} in the FU samples.

Spiked concentration	30 μg kg^{-1}		120 μg kg^{-1}		240 μg kg^{-1}	
Pesticides	Recovery(%)	RSD(%)	Recovery(%)	RSD(%)	Recovery(%)	RSD(%)
Acephate	76.3	0.7	88.1	2	82.6	2
Acetamiprid	75.2	0.8	85.3	2	80.3	3
Alachlor	95.1	3	99.4	2	97.7	1
Atrazine	97.6	0.6	97.8	2	96.9	3
Avermectin	55.5	19	69.6	12	61.3	3
Azaconazole	86.5	4	102	3	98.9	1
Azamethiphos	79.0	9	92.6	1	84.0	4
Azoxystrobin	89.6	6	98.7	2	91.0	5
Benalaxyl	95.5	4	118	3	92.9	1
Bendiocarb	101	4	92.9	3	103	5
Bitertanol	79.5	12	81.2	7	87.8	8
Bromuconazole	87.8	6	99.3	1	98.1	4
Buprofezin	84.3	2	92.1	0.5	88.3	5
Cadusafos	103	4	111	3	97.0	6
Carbendazim	77.2	6	57.3	5	45.6	5
Carbofuran	92.6	10	97.2	4	92.3	2
Chlorantraniliprole	66.9	13	63.0	1	68.2	6
Chlorpyrifos	77.5	2	85.4	3	81.1	5
Chlortoluron	62.4	4	75.2	2	73.7	4
Clothianidin	76.7	8	86.6	2	86.2	0.4
Coumaphos	35.5	9	51.4	7	48.7	6
Cyanofenphos	109	12	106	1	97.5	6
Cyazofamid	98.8	8	98.4	3	98.6	2
Cyproconazole	95.6	3	106	3	98.0	4

Dicrotophos	93.3	3	96.5	2	96.7	2
Difenoconazole	96.9	6	90.7	3	109	8
Dimethoate	75.8	4	85.4	0.3	86.7	3
Dimethomorph	91.0	2	97.5	4	96.1	1
Dimethyl phthalate	88.6	3	82.2	6	101	5
Diniconazole	95.5	6	79.3	3	103	9
Dinotefuran	90.5	5	97.9	5	91.0	3
Emamectin Benzoate	36.7	10	41.7	5	36.2	12
Epoxiconazol	89.6	4	75.2	2	76.4	0.7
Ethiofencarb	88.6	2	89.6	1	91.4	4
Ethofenprox	47.5	7	52.9	2	53.7	12
Etoxazole	89.5	1	98.1	2	95.1	3
Fenamiphos	91.0	4	95.0	3	92.2	5
Fenamiphos sulfoxide	91.2	4	97.4	2	101	2
Fenbuconazole	80.7	5	87.6	3	84.3	6
Fenpropathrin	90.8	13	80.3	2	88.2	5
Fipronil	171	21	94.4	1	91.3	1
Fipronil-sulfone	90.0	3	109	1	94.8	2
Fludioxonil	61.8	10	77.2	6	82.2	2
Fluopyram	91.0	3	112	0.4	102	5
Fluoxastrobin	92.4	6	119	2	101	4
Flusilazole	95.9	6	105	4	104	0.9
Flutolanil	95.3	6	118	3	99.2	1
Flutriafol	94.4	8	94.3	2	103	5
Hexaconazole	97.5	9	114	0.3	96.4	9
Hexaflumuron	84.7	2	110	2	106	4
Hexazinone	91.4	4	94.7	4	99.4	3
3-Hydroxy-carbofuran	88.2	7	75.5	4	85.3	7

Imazalil	67.8	8	80.0	8	71.9	2
Imidacloprid	86.9	6	92.4	7	86.5	4
Indoxacarb	88.3	6	116	3	104	6
Ipconazole	69.2	5	87.3	6	81.4	3
Isoprocarb	97.6	3	97.2	6	99.4	4
Kresoxim-methyl	78.0	4	103	1	107	5
Lufenuron	34.3	5	49.5	2	55.0	12
Machette	90.5	4	102	0.2	100	0.8
Malaoxon	101	2	96.3	2	98.1	5
Malathion	104	3	110	2	102	3
Mepronil	82.7	4	96.6	4	97.3	1
Metalaxyl	97.5	6	97.6	3	95.8	3
Metalaxyl-M	93.2	5	99.7	2	98.5	3
Methidathion	104	12	91.9	2	97.1	4
Methomyl	72.3	9	85.6	2	83.5	5
Methoprene	106	17	95.0	3	88.3	9
Methoxyfenozide	84.3	9	106	4	99.6	2
Mevinphos	75.2	9	86.0	1	83.0	4
Monocrotophos	92.5	6	88.1	1	96.1	4
Myclobutanil	94.9	2	103	2	98.0	2
Omethoate	85.0	3	96.5	2	88.3	2
Paclobutrazol	96.4	5	110	7	93.6	0.9
Penconazole	94.1	5	114	1	93.3	3
Phenthoate	86.1	3	106	1	96.2	4
Phorate	109	8	127	6	95.6	0.8
Phorate sulfone	81.3	3	99.2	3	100	2
Phosalone	64.2	3	90.8	4	80.5	7
Phosfolan	88.0	3	88.5	3	92.5	7

Phosphamidon	92.4	8	96.6	7	91.9	6
Piperonyl butoxide	76.2	3	93.9	0.4	87.8	8
Pirimicarb	87.9	1	92.8	4	99.3	3
Pirimiphos-methyl	93.6	2	121	4	105	4
Prochloraz	38.4	7	59.2	9	52.5	7
Profenofos	80.5	5	85.7	3	84.3	3
Propamocarb	65.1	4	70.8	3	66.6	1
Propiconazole	87.3	6	115	2	92.1	3
Pyraclostrobin	35.2	9	37.9	6	39.1	11
Pyridaben	91.3	3	91.9	3	91.0	1
Pyridafenthion	75.3	0.7	99.7	4	100	8
Pyrimitate	94.9	5	97.9	2	95.4	4
Pyrisoxazole	94.3	7	80.1	2	82.9	5
Simeconazole	95.0	3	113	2	112	3
Spirodiclofen	76.3	10	86.8	3	91.0	4
Sulfotep	94.7	4	109	3	108	3
Sulprofos	83.6	8	87.9	5	96.3	2
Tebuconazole	91.2	5	97.8	2	77.1	6
Tebufenozide	92.2	0.3	110	4	112	3
Tebufenpyrad	87.3	1	95.4	3	96.4	4
Temephos	68.6	3	75.7	6	79.3	6
Terbufos	108	6	98.8	8	96.3	5
Tetrachlorvinphos	86.8	2	99.6	3	95.1	2
Tetraconazole	97.7	6	107	3	108	2
Thiacloprid	70.8	7	81.0	2	78.7	4
Thiamethoxam	82.4	3	85.6	3	84.6	2
Thiophanate-methyl	72.5	4	73.2	4	72.9	2
Tolfenpyrad	46.1	13	52.0	6	59.8	11

Triadimefon	96.6	4	108	2	95.2	2
Triadimenol	88.7	7	106	2	98.0	0.9
Trifloxystrobin	97.8	1	119	2	117	14
Triticonazole	85.3	4	99.1	3	96.2	1
Uniconazole	93.0	4	102	2	93.6	2
Vitavax	77.3	5	78.4	3	92.2	0.5

Table S8 Recoveries of the pesticides at spiked concentrations of 30, 120, and 240 μg kg^{-1} in the FC samples.

Spiked concentration	30 μg kg^{-1}		120 μg kg^{-1}		240 μg kg^{-1}	
Pesticides	Recovery(%)	RSD(%)	Recovery(%)	RSD(%)	Recovery(%)	RSD(%)
Acephate	76.6	4	82.5	2	88.3	3
Acetamiprid	84.5	6	87.0	4	95.8	3
Alachlor	106	2	95.8	3	97.2	2
Atrazine	95.1	8	97.1	2	101	5
Avermectin	83.0	7	84.0	12	77.5	7
Azaconazole	93.7	5	92.6	1	97.8	3
Azamethiphos	66.3	5	92.5	0.02	82.5	2
Azoxystrobin	93.1	4	95.2	2	95.6	4
Benalaxyl	94.8	5	97.3	0.6	102	11
Bendiocarb	84.7	9	92.2	11	97.7	2
Bitertanol	112	10	91.8	9	89.9	8
Bromuconazole	78.6	11	101	5	94.8	2
Buprofezin	93.3	2	93.4	2	90.5	5
Cadusafos	103	1	98.3	2	96.5	5
Carbendazim	18.7	6	22.7	3	21.7	9
Carbofuran	78.8	10	89.5	5	101	1
Chlorantraniliprole	60.9	11	64.5	6	57.9	5
Chlorpyrifos	77.0	5	82.8	3	84.0	6
Chlortoluron	63.0	3	63.5	9	78.3	0.3
Clothianidin	67.3	6	83.1	2	85.9	4
Coumaphos	38.0	9	38.3	10	40.6	9
Cyanofenphos	90.8	4	97.2	1	92.0	1
Cyazofamid	116	8	88.8	3	93.1	5
Cyproconazole	95.8	4	95.4	4	94.8	3

Dicrotophos	93.2	7	96.5	0.9	96.9	2
Difenoconazole	81.0	2	83.8	4	80.1	0.9
Dimethoate	81.6	3	87.6	1	102	0.6
Dimethomorph	93.0	0.5	93.4	4	90.8	2
Dimethyl phthalate	90.8	9	90.8	3	89.0	4
Diniconazole	77.5	7	78.1	3	74.0	0.3
Dinotefuran	88.4	3	100	2	91.3	2
Emamectin Benzoate	43.3	11	38.2	10	35.3	10
Epoxiconazol	107	2	78.3	3	83.7	2
Ethiofencarb	88.0	6	88.6	4	97.6	2
Ethofenprox	62.9	11	56.4	9	87.5	10
Etoxazole	90.7	1	90.3	0.9	95.0	2
Fenamiphos	95.9	3	97.2	2	95.2	4
Fenamiphos sulfoxide	77.9	2	90.4	9	98.4	10
Fenbuconazole	102	5	78.6	2	90.5	4
Fenpropathrin	80.1	4	86.3	9	88.8	11
Fipronil	152	22	95.5	6	99.3	2
Fipronil-sulfone	94.9	0.8	103	1	97.9	0.6
Fludioxonil	83.9	13	70.6	9	72.1	3
Fluopyram	94.8	2	95.3	1	98.7	2
Fluoxastrobin	92.9	7	101	1	95.3	3
Flusilazole	93.7	6	96.1	2	93.8	2
Flutolanil	90.4	0.9	96.7	3	101	2
Flutriafol	88.7	10	91.0	5	96.2	4
Hexaconazole	100	1	98.1	4	97.4	6
Hexaflumuron	91.6	1	94.8	2	94.0	0.9
Hexazinone	76.7	4	89.3	2	96.0	3
3-Hydroxy-carbofuran	78.9	3	85.2	2	106	11

Imazalil	71.8	2	81.3	5	74.5	4
Imidacloprid	70.1	11	93.5	6	86.2	4
Indoxacarb	82.0	5	103	9	97.9	2
Ipconazole	72.6	6	78.5	1	84.0	2
Isoprocarb	97.2	2	89.4	3	98.2	3
Kresoxim-methyl	100	7	97.4	4	102	0.5
Lufenuron	37.3	3	44.3	10	47.4	13
Machette	96.6	0.4	96.4	2	98.5	2
Malaoxon	92.6	4	98.7	3	101	8
Malathion	96.7	4	94.2	2	95.7	3
Mepronil	80.2	2	80.9	2	95.4	0.7
Metalaxyl	88.0	5	101	3	95.9	4
Metalaxyl-M	87.9	3	97.5	2	96.6	0.7
Methidathion	88.4	11	80.4	0.6	93.0	5
Methomyl	89.4	4	95.0	4	99.5	2
Methoprene	89.1	10	91.2	5	97.4	5
Methoxyfenozide	94.3	4	91.4	3	97.5	5
Mevinphos	88.2	6	88.7	8	94.1	6
Monocrotophos	94.6	3	95.8	4	97.9	0.03
Myclobutanil	102	3	98.5	7	107	4
Omethoate	78.0	4	92.1	4	92.9	2
Paclobutrazol	95.2	1	98.1	1	99.3	4
Penconazole	93.2	8	97.1	0.7	93.2	6
Phenthoate	96.1	6	99.9	2	98.5	4
Phorate	71.4	3	105	4	101	0.9
Phorate sulfone	98.8	6	95.2	2	105	7
Phosalone	72.2	4	71.8	6	72.9	7
Phosfolan	89.6	8	95.2	7	97.9	2

Phosphamidon	93.6	7	98.5	6	104	5
Piperonyl butoxide	91.1	1	85.4	2	86.9	3
Pirimicarb	92.7	6	89.5	2	99.5	1
Pirimiphos-methyl	93.8	2	97.8	3	98.5	2
Prochloraz	42.7	10	50.6	12	46.6	3
Profenofos	82.9	0.6	88.5	3	82.7	4
Propamocarb	62.5	0.5	78.6	1	76.4	3
Propiconazole	98.8	2	91.7	3	94.7	2
Pyraclostrobin	34.4	2	30.8	8	34.8	7
Pyridaben	90.0	2	93.8	1	95.9	9
Pyridafenthion	89.9	8	86.1	1	91.6	3
Pyrimitate	94.0	2	92.6	1	98.9	4
Pyrisoxazole	83.3	9	72.4	3	74.0	4
Simeconazole	108	7	98.0	3	103	2
Spirodiclofen	92.9	7	90.5	2	97.8	3
Sulfotep	96.2	1	103	1	101	3
Sulprofos	86.4	3	87.0	3	98.7	7
Tebuconazole	92.4	7	90.7	1	88.0	4
Tebufenozide	99.2	8	106	8	93.2	1
Tebufenpyrad	89.2	7	92.5	6	96.4	4
Temephos	78.5	5	73.5	2	76.3	7
Terbufos	109	4	99.1	10	98.4	2
Tetrachlorvinphos	102	8	99.9	5	95.1	1
Tetraconazole	100	6	98.7	0.4	105	2
Thiacloprid	79.0	1	87.1	3	96.7	5
Thiamethoxam	96.0	3	97.9	2	96.2	2
Thiophanate-methyl	67.8	3	70.3	5	90.8	5
Tolfenpyrad	59.1	12	57.0	14	54.4	8

Triadimefon	108	4	99.1	2	96.5	3
Triadimenol	92.3	3	103	5	104	4
Trifloxystrobin	91.9	1	96.5	2	98.0	4
Triticonazole	96.5	7	96.2	4	96.1	1
Uniconazole	92.4	2	94.0	2	93.6	3
Vitavax	69.6	4	79.1	3	86.4	6

Table S9 Recoveries of the pesticides at spiked concentrations of 30, 120, and 240 μg kg^{-1} in the FD samples.

Spiked concentration	30 μg kg^{-1}		120 μg kg^{-1}		240 μg kg^{-1}	
Pesticides	Recovery(%)	RSD(%)	Recovery(%)	RSD(%)	Recovery(%)	RSD(%)
Acephate	74.6	3	82.6	5	86.4	2
Acetamiprid	92.6	4	87.0	4	78.9	6
Alachlor	97.0	0.9	94.4	0.4	94.3	1
Atrazine	110	4	93.7	1	89.4	3
Avermectin	89.6	13	68.9	8	73.4	0.9
Azaconazole	94.3	8	95.6	1	96.4	2
Azamethiphos	84.7	0.2	87.2	9	62.6	6
Azoxystrobin	92.5	5	91.8	6	88.2	0.7
Benalaxyl	96.5	4	94.1	3	98.0	8
Bendiocarb	96.0	9	77.8	3	72.9	5
Bitertanol	114	11	93.5	4	91.9	4
Bromuconazole	100	8	90.3	2	88.8	6
Buprofezin	95.9	3	97.6	0.7	91.5	3
Cadusafos	103	3	94.6	0.3	93.5	6
Carbendazim	31.5	3	27.9	9	28.9	6
Carbofuran	98.8	7	81.9	8	77.8	5
Chlorantraniliprole	86.4	11	62.7	3	80.0	7
Chlorpyrifos	93.4	1	86.6	4	87.1	3
Chlortoluron	78.7	5	71.7	5	71.3	3
Clothianidin	96.5	10	88.9	0.7	91.3	6
Coumaphos	47.3	4	49.3	8	56.7	2
Cyanofenphos	98.2	5	96.3	6	99.5	7
Cyazofamid	91.6	17	85.5	2	97.9	7
Cyproconazole	104	4	95.6	2	94.5	3

Dicrotophos	93.5	4	103	1	95.9	2
Difenoconazole	82.1	5	82.1	6	80.4	6
Dimethoate	95.2	5	84.9	2	85.0	1
Dimethomorph	92.6	4	94.4	2	95.0	5
Dimethyl phthalate	86.0	10	74.5	3	66.3	9
Diniconazole	91.6	6	80.0	8	81.0	6
Dinotefuran	94.8	4	95.2	3	94.7	2
Emamectin Benzoate	49.9	2	37.8	11	50.9	7
Epoxiconazol	100	3	82.1	1	83.3	6
Ethiofencarb	98.5	10	84.1	4	94.1	9
Ethofenprox	72.9	0.9	60.1	4	64.6	4
Etoxazole	91.3	5	92.2	3	96.0	1
Fenamiphos	96.2	8	94.9	4	96.6	6
Fenamiphos sulfoxide	102	10	83.2	4	70.4	2
Fenbuconazole	90.8	9	82.5	2	82.4	6
Fenpropathrin	97.2	4	90.8	8	88.5	4
Fipronil	98.4	16	99.8	6	77.3	11
Fipronil-sulfone	99.6	2	102	0.9	93.1	0.8
Fludioxonil	63.3	11	76.8	2	80.8	9
Fluopyram	99.8	2	98.5	3	99.8	2
Fluoxastrobin	95.3	9	96.7	3	97.6	2
Flusilazole	94.2	8	97.3	4	96.8	3
Flutolanil	97.9	6	94.5	2	97.3	4
Flutriafol	113	6	91.7	5	93.1	4
Hexaconazole	98.9	6	91.6	1	96.9	5
Hexaflumuron	98.2	5	97.8	3	99.5	2
Hexazinone	90.0	7	76.8	6	73.6	4
3-Hydroxy-carbofuran	95.0	19	95.0	1	88.3	7

Imazalil	65.6	4	76.9	5	79.2	0.9
Imidacloprid	99.8	9	93.2	0.4	89.4	2
Indoxacarb	117	6	98.5	3	92.0	8
Ipconazole	87.4	3	77.6	2	82.9	7
Isoprocarb	106	5	102	3	91.7	3
Kresoxim-methyl	104	7	90.9	0.2	96.3	0.8
Lufenuron	64.2	7	52.1	4	70.6	1
Machette	97.5	2	97.6	0.3	95.2	2
Malaoxon	92.6	8	72.1	3	71.5	8
Malathion	98.8	3	96.0	1	96.8	5
Mepronil	87.8	4	83.3	2	94.5	0.8
Metalaxyl	103	4	98.8	3	97.9	7
Metalaxyl-M	111	3	97.7	3	94.1	2
Methidathion	108	0.5	92.2	4	93.9	6
Methomyl	93.0	4	101	1	89.9	2
Methoprene	103	2	91.2	5	91.8	3
Methoxyfenozide	97.9	8	94.5	3	100	6
Mevinphos	94.8	13	92.4	6	83.8	7
Monocrotophos	95.1	5	82.9	5	93.9	0.4
Myclobutanil	105	9	97.4	1	95.3	7
Omethoate	86.5	5	89.6	2	89.2	2
Paclobutrazol	109	3	95.0	5	97.2	3
Penconazole	91.1	5	96.4	5	93.2	4
Phenthoate	108	7	101	4	95.8	4
Phorate	91.7	5	95.3	2	94.5	7
Phorate sulfone	110	6	90.3	1	99.9	4
Phosalone	75.1	5	73.3	2	78.5	6
Phosfolan	99.1	4	88.9	2	78.6	2

Phosphamidon	84.2	3	91.1	3	91.1	4
Piperonyl butoxide	97.6	5	93.4	3	91.3	3
Pirimicarb	93.8	8	92.3	6	89.7	3
Pirimiphos-methyl	95.0	7	96.8	3	91.3	4
Prochloraz	60.4	0.6	51.9	4	59.2	5
Profenofos	94.4	0.6	83.9	4	90.8	3
Propamocarb	63.8	5	70.6	8	70.1	0.6
Propiconazole	94.8	4	95.9	2	93.0	2
Pyraclostrobin	48.4	5	40.6	3	51.8	1
Pyridaben	95.5	2	92.0	2	90.2	3
Pyridafenthion	87.1	7	90.5	4	97.8	4
Pyrimitate	98.8	5	98.9	2	96.8	2
Pyrisoxazole	81.9	15	81.0	4	79.7	3
Simeconazole	94.7	0.5	96.6	5	98.6	2
Spirodiclofen	83.3	3	90.3	4	98.9	1
Sulfotep	118	2	98.6	0.5	95.9	2
Sulprofos	91.8	5	86.3	2	96.1	4
Tebuconazole	91.7	2	91.7	4	88.7	8
Tebufenozide	108	9	95.9	0.8	97.8	4
Tebufenpyrad	98.0	6	92.0	3	92.8	2
Temephos	87.7	3	76.7	2	83.8	3
Terbufos	95.7	7	88.9	4	93.3	5
Tetrachlorvinphos	106	10	97.0	0.7	94.5	8
Tetraconazole	106	1	106	2	94.3	3
Thiacloprid	86.3	4	80.9	2	78.2	0.8
Thiamethoxam	104	2	97.7	2	94.3	3
Thiophanate-methyl	74.9	3	62.7	2	61.1	3
Tolfenpyrad	70.0	0.7	63.2	4	68.0	1

Triadimefon	102	11	94.0	2	94.8	8
Triadimenol	100	12	95.2	3	95.7	2
Trifloxystrobin	104	3	98.6	1	91.3	4
Triticonazole	110	17	99.4	6	97.5	5
Uniconazole	106	2	92.2	3	91.4	5
Vitavax	90.7	6	70.4	5	83.5	3

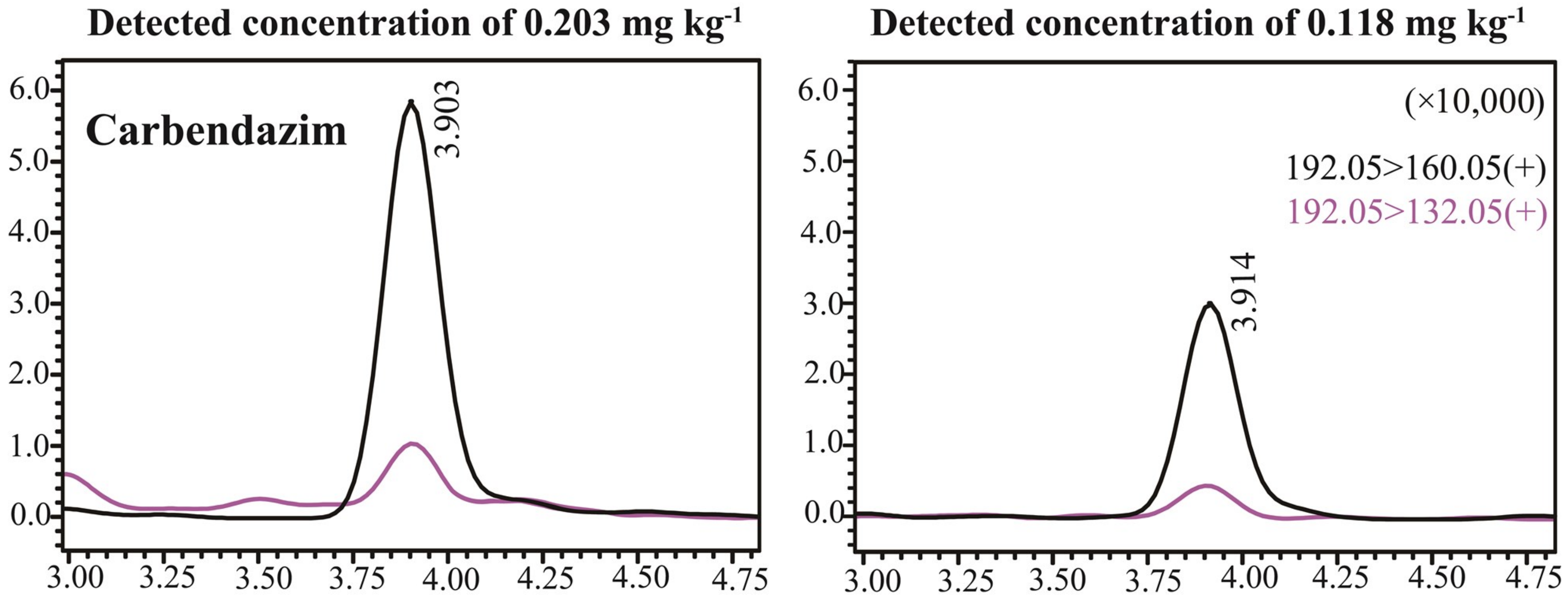

Fig. S1 LC-MS/MS chromatograms of detected carbendazim at concentrations exceeding the EU MRLs in two real samples.
